# Optimizing exercise modalities to enhance brain-derived neurotrophic factor levels in older adults: a Bayesian network and dose-response meta-analysis

**DOI:** 10.3389/fphys.2026.1777058

**Published:** 2026-07-15

**Authors:** Guangwen Liu, Zhide Liang, Wance Wang, Renkai Ge

**Affiliations:** 1 School of Physical Education and Health, East China Jiaotong University, Nanchang, China; 2 Faculty of Health Sciences and Sports, Macao Polytechnic University, Rua de Gomes, Macau, China

**Keywords:** BDNF, dose-response, exercise, meta-analysis, neuroplasticity, older adults

## Abstract

**Background:**

Global aging leads to increased age-related cognitive decline and neurodegenerative diseases like dementia. Brain-derived neurotrophic factor (BDNF) is crucial for neuronal survival, synaptic plasticity, and cognitive function, but its levels often decline with age. Physical exercise can potentially elevate BDNF levels in older adults. Previous meta-analyses had limitations in comparing diverse exercise types simultaneously and exploring dose-response relationships.

**Objective:**

To comprehensively compare the relative effects of exercise types on BDNF levels in older adults and explore dose-response relationships using a multilevel Bayesian network meta-analysis.

**Methods:**

A systematic search of PubMed, Embase, Web of Science, and the Cochrane Library for randomized controlled trials (RCTs) involving exercise interventions in older adults was conducted until April 2025. Data on BDNF levels, exercise type, duration, frequency, and intensity were collected. A multilevel Bayesian network meta-analysis was performed to compare exercise interventions and explore dose-response relationships.

**Results:**

Forty-seven RCTs with 1815 participants were included. Aquatic exercise showed the largest point estimate (SMD = 1.00), followed by mind-body exercise (SMD = 0.61) and mixed exercise (SMD = 0.30). However, the AQE estimate was based on sparse evidence and should be interpreted cautiously. Several exercise modalities exceeded a 0.5-SD distribution-based interpretability threshold. Aerobic, combined aerobic and resistance, and resistance exercises also improved BDNF levels, whereas HIIT remained uncertain in the primary analysis, although the active-comparator-exclusion sensitivity analysis yielded a positive estimate that requires confirmation. Dose-response analyses showed non-linear relationships, with effects observed across dosages and after intervention cessation. Advanced age was linked to diminished effects.

**Conclusion:**

Several exercise modalities may increase BDNF levels in older adults, but AQE and HIIT should be interpreted as preliminary or hypothesis-generating. Complex dose-response relationships exist, and age and sex may moderate effectiveness. Treatment rankings should be interpreted cautiously in light of evidence certainty.

## 1 Introduction

The aging of the global population is significant. By 2050, the number of individuals aged ≥60 years worldwide will increase to 2.1 billion, accounting for 22% of the total population (United Nations, 2019), implying that, one in every six individuals will be aged >60 years by 2050. Simultaneously, the incidences of age-related cognitive decline and neurodegenerative diseases, such as dementia, are continuously increasing, posing a severe challenge to public health. Currently, more than 55 million individuals worldwide have dementia, with nearly 10 million new cases emerging each year (Alzheimer's, D. I, 2024). The number of patients is projected to reach 139 million by 2050, creating immense socioeconomic burden and caregiving pressure (Alzheimer's, D. I, 2024). Brain-derived neurotrophic factor (BDNF), a key neurotrophic factor, plays a crucial role in maintaining neuronal survival, promoting synaptic plasticity, and supporting cognitive functions such as learning and memory (Lu et al., 2014). However, BDNF levels often decline with age, which is closely associated with cognitive decline in older adults and an increased risk of neurodegenerative diseases (Lommatzsch et al., 2005). Therefore, exploring effective strategies to delay this decrease or improve BDNF levels in older populations is of significant public health importance for maintaining brain health and preventing cognitive impairment.

Given the core role of BDNF in delaying age-related cognitive decline (Numakawa and Odaka, 2022), various intervention strategies have been explored to enhance or maintain BDNF levels in the older population. These strategies include cognitive training, mindfulness practices, specific dietary nutritional supplements, and environmental enrichment (Leon and Woo, 2018; Nicastri et al., 2022; Sohouli et al., 2023). However, these non-exercise interventions may face limitations in practical application, such as patient adherence, cost-effectiveness, generalizability, inconsistent effects, or lack of sufficient evidence (Duraney et al., 2025; Lampit et al., 2014). Against this backdrop, physical exercise, a low-cost, easily promotable, and non-pharmacological intervention with broad health benefits, has garnered attention owing to its potential to improve brain health and increase BDNF levels (Szuhany et al., 2015; Walsh et al., 2020). Regular physical exercise, particularly certain specific types of movement, can effectively promote BDNF secretion in older adults, thereby positively influencing neuroprotection and cognitive function (Dai et al., 2024; Farrukh et al., 2023; Marinus et al., 2019).

The effect of exercise on BDNF levels in older populations has been investigated in several meta-analyses (Dinoff et al., 2016; Gholami et al., 2025; Marinus et al., 2019; Setayesh and Mohammad Rahimi, 2023; Wang et al., 2020). However, these studies had some limitations. First, most of these previously published meta-analyses were limited to traditional pairwise meta-analyses, comparing specific types of exercise to a control group, which makes it difficult to comprehensively assess and rank the relative effects of different types of exercise simultaneously (Dinoff et al., 2016; Gholami et al., 2025; Marinus et al., 2019; Setayesh and Mohammad Rahimi, 2023; Wang et al., 2020). Second, the results of some studies were inconsistent. For instance, Dinoff et al. (2016) suggested that aerobic exercise had a positive effect, whereas resistance exercise was not clearly effective, while Marinus et al. (2019) reported positive effects of resistance exercise in older adults. Furthermore, when original studies include multiple exercise intervention groups, traditional analytical methods may not adequately account for the inherent correlations and dependencies among different comparisons within these multi-arm studies. This could affect the accuracy and robustness of pooled effect estimates (Caldwell et al., 2005). More importantly, previous meta-analyses provided limited in-depth exploration of the dose-response relationship between exercise and BDNF levels (Wang et al., 2020; Setayesh and Mohammad Rahimi, 2023; Marinus et al., 2019; Gholami et al., 2025; Dinoff et al., 2016), which is crucial for formulating optimal exercise prescriptions to maximize their neuroprotective benefits. Therefore, current research urgently requires an analytical approach capable of simultaneously comparing multiple interventions, properly handling complex data structures and exploring dose-response relationships.

Therefore, this study employed a multilevel Bayesian network meta-analysis (NMA) approach. It aimed to comprehensively compare and rank the relative effects of different types of exercise on BDNF levels in older adults, while also conducting an in-depth exploration of the dose-response relationships between key exercise parameters and BDNF effects. This study aimed to provide comprehensive evidentiary support for developing optimized exercise prescriptions to promote brain health in older adults.

## 2 Methods

### 2.1 Registration

This meta-analysis adheres to the Cochrane Handbook for Systematic Reviews of Interventions (Cumpston et al., 2023) and followed the Preferred Reporting Items for Systematic Reviews and Meta-Analyses extension statement for NMAs (Page et al., 2021; Hutton et al., 2015). This study has been registered in PROSPERO (CRD420251048624).

### 2.2 Search strategy

**Figure 1:**
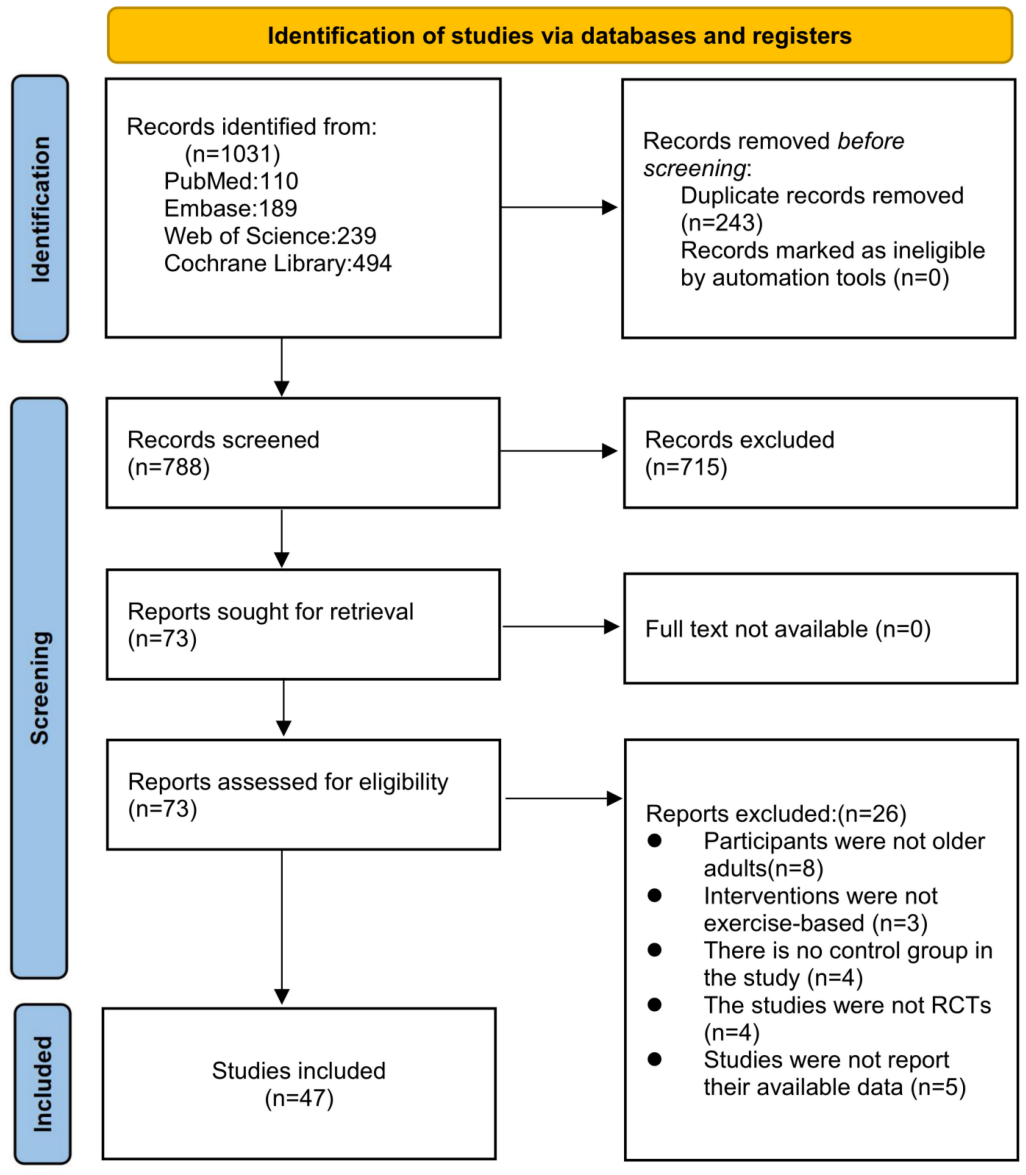
Literature review flowchart. RCTs, randomized controlled trials.

We searched PubMed, Embase, Web of Science, and Cochrane Library from their inception to April 2025. The search strategy combined Medical Subject Headings and free-text words, focusing on three core concepts: “older adults,” “various exercise interventions” (including aerobic exercise, resistance training, mind-body exercise, and comprehensive exercise), and “brain-derived neurotrophic factor.” To capture resistance-oriented interventions across databases, the search strategy used broad exercise-related controlled vocabulary and free-text terms, including exercise training, strength-related terms, and database-specific terminology; reference lists of included studies and relevant reviews were also manually screened to reduce the risk of missing eligible studies, including resistance-training trials. No language restrictions were applied to any searches to ensure comprehensive coverage of the literature. The detailed literature screening process is illustrated in **Figure 1**.

### 2.3 Eligibility criteria

The inclusion criteria for this NMA were structured according to the PICO framework: Population, older adults; Intervention, eligible exercise modalities; Comparator, eligible comparator/control conditions or other eligible exercise interventions; and Outcome, circulating BDNF level. The inclusion criteria were as follows:

#### 2.3.1 Population

Older individuals were included in this study. Although the age threshold for “older adults” varies in the literature, commonly referring to individuals aged 60 or ≥65 years, for this NMA, the operational inclusion criterion was set to participants aged ≥55 years to include a broader range of relevant studies. Study participants included older adults living in the community, nursing homes, or hospitals, who were healthy older adults or had stable chronic non-communicable diseases (such as hypertension, type 2 diabetes mellitus, or mild cognitive impairment) and could participate in the listed exercise interventions.

#### 2.3.2 Interventions

Studies were considered for inclusion if they included at least one of the following clearly defined exercise intervention: aerobic exercise (AE), combined aerobic and resistance exercise (AE + RE), aquatic exercise (AQE), coordination exercise (CE), high-intensity interval training (HIIT), mind-body exercise (MBE, such as Tai Chi, Yoga, or Qigong), mixed exercise (ME, a combination of three or more different exercise forms listed above), or resistance exercise (RE).

#### 2.3.3 Comparisons

Studies were included if they involved at least one eligible exercise intervention or comparator condition. Comparator/control conditions were classified according to the full-text descriptions as inactive control, usual care/standard treatment, active non-exercise control, active physical control, or no non-exercise control arm. Studies without a non-exercise control arm contributed exercise–exercise comparisons and were not treated as an additional comparator node. The full classification is provided in **Supplementary Table S3**.

#### 2.3.4 Outcomes

The primary outcome was BDNF level.

#### 2.3.5 Study design

Only randomized controlled trials (RCTs) were included. This encompassed parallel-group design, crossover design (only data from the first period or data ensuring no carry-over effect were included), and cluster-randomized trials.

Studies were excluded if they met any of the following criteria:

1. Exercise was not the primary or independent intervention factor in the study, the exercise intervention was poorly defined or could not be categorized, or the duration of the intervention did not meet the pre-set minimum standard.
2. Acute effect studies: Studies that only assessed the immediate effects of a single exercise session.
3. Data or publication issues: Duplicate publications (the one with the most complete data was selected), conference-only reports, unpublished datasets, author-supplied unpublished data, and literature for which full text or key data could not be obtained were excluded.

### 2.4 Study selection

All retrieved literature records were imported into EndNote X9 software and duplicates were removed. Subsequently, two researchers [Gw-L and Zd-L] independently screened the titles and abstracts of the literature according to the pre-defined inclusion and exclusion criteria, followed by a full-text review of potentially eligible articles. Any disagreements during the screening process were resolved through discussion or by a third author [Rk-G] if consensus could not be reached. All quantitative data used in this review were extracted from published full-text RCT reports and their published supplementary materials where applicable. No unpublished individual participant data, conference-only data, author-supplied unpublished datasets, or original unpublished data were used. Effect sizes were calculated from published aggregate summary statistics.

### 2.5 Data extraction and code management

Two researchers [Gw-L and Zd-L] independently extracted key information from each included study using a predesigned standardized data extraction form. It included basic study characteristics (author, year, and region), participant characteristics (sample size, age, sex, and health status), details of the exercise interventions for each group (type of exercise, duration per session, weekly frequency, total intervention period, and reported intensity), specific details of the control group, and BDNF data (mean, standard deviation, sample size at baseline and post-intervention, measurement unit, and sample source). Any disagreements during the extraction process were resolved through discussion, with the third author [Rk-G] making the final decision if necessary. To quantify and uniformly compare the exercise intervention dosage across different studies, we coded the extracted exercise data: metabolic equivalents (METs) per min for each activity were estimated by referencing the “2024 Adult Compendium of Physical Activities (Herrmann et al., 2024).” This, combined with the duration per session and weekly frequency, was used to calculate the total weekly exercise dose (MET-min/week = MET value per min × duration per session × weekly exercise frequency). This coding process was completed independently by two researchers [Gw-L and Zd-L] and cross-checked to ensure accuracy. Comparator/control descriptions were additionally re-extracted from the full-text reports by two reviewers (Gw-L and Zd-L), including original wording, source section, final category, active-comparator status, and classification rationale. Disagreements were resolved through discussion with a third reviewer (Rk-G). The study-level classification is presented in **Supplementary Table S3**.

### 2.6 Risk of bias and confidence assessment

The risk of bias for the BDNF outcome in all included RCTs was independently assessed by two researchers [Gw-L and Zd-L] using the revised Cochrane Risk of Bias tool for randomized trials (RoB 2.0) (**Appendix A3**, **Supplementary Figures S1**, **S2**) (Sterne et al., 2019). Each study was judged as “low risk,” “some concerns,” or “high risk” across five domains: bias arising from the randomization process, bias due to deviations from intended interventions, bias due to missing outcome data, bias in measurement of the outcome, and bias in selection of the reported result. Subsequently, the same two researchers independently evaluated the confidence in the evidence for each comparison in this NMA using the Confidence in Network Meta-Analysis (CINeMA) framework (Nikolakopoulou et al., 2020). This evaluation process systematically considered six domains: within-study bias, reporting bias, indirectness, imprecision, heterogeneity/inconsistency, and incoherence. Based on a comprehensive judgment of these domains, the confidence in evidence for each comparison was rated as “high,” “moderate,” “low,” or “very low.”

### 2.7 Measures of treatment effect

This study used Hedges’ g (standardized mean difference [SMD]) as the primary effect size measure to compare the effects of different exercise interventions on BDNF levels (Hedges and Olkin, 2014). Because BDNF was reported using different biological sample sources, assay procedures, and measurement units across studies, standardized mean differences were used to place effects on a common scale; however, this approach cannot fully eliminate methodological heterogeneity in BDNF quantification. The effect size was calculated based on the mean post-intervention change in BDNF level or the change relative to baseline for each group, along with the corresponding standard deviation (SD) and sample size. If the included studies did not directly report the SD, it was estimated using standard statistical methods from the provided standard errors, 95% confidence intervals (CIs) of the mean, or medians and interquartile ranges (Borenstein et al., 2021). While calculating effect sizes based on change-from-baseline values, we estimated the required SDs of change scores using an assumed pre–post correlation coefficient when the SDs of change scores were not directly reported, as recommended in the Cochrane Handbook (Cumpston et al., 2023). The primary analysis used a moderate correlation coefficient of r = 0.5. Because the true pre–post correlation may vary across exercise modalities, intervention durations, baseline fitness, participant characteristics, and measurement conditions, we additionally conducted sensitivity analyses using r = 0.2 and r = 0.8 to assess the robustness of the calculated Hedges’ g values and network estimates.

### 2.8 Statistical analysis

We employed a multilevel Bayesian NMA to comprehensively evaluate and compare the relative effects of different exercise interventions on BDNF levels in older adults (Hox et al., 2017). This analysis was primarily executed using R statistical software (version 4.4.3) and its “brms” package, which utilizes the “Stan” language for backend Bayesian inference (Bürkner, 2017). The specific model structure was smd | se(se_smd) ~ 1 + trt + (1 | studyID), where trt represents different types of exercise interventions (fixed effect) and (1 | studyID) represents the study-level (study ID) random effect (random intercept). This random effect was used to account for and quantify heterogeneity (represented by the between-study variance τ^2^) arising from clinical or methodological differences across studies (Gelman et al., 2013; Higgins et al., 2009). The primary analysis retained a pragmatic broad-comparator reference node to preserve network connectivity; however, this node was not interpreted as implying clinical equivalence among all comparator conditions. This modelling approach allows the simultaneous estimation of relative treatment effects for all possible pairwise comparisons within the network (Caldwell et al., 2005). Within the Bayesian framework, we specified prior distributions for the model parameters. Specifically, weakly informative or non-informative normal (0, σ = 1) priors were assigned to the fixed effects (Gelman, 2006; Gelman et al., 2013). Markov Chain Monte Carlo (MCMC) simulations were conducted by running eight independent chains, each with 10,000 iterations, where the first 5,000 iterations were discarded as a warm-up (burn-in) period to ensure sampling from the stationary posterior distribution of the parameters (Gelman et al., 2013). Convergence of the MCMC chains was assessed by examining the mixing of trace plots, analyzing the Potential Scale Reduction Factor (R-hat, which should be close to 1.0), and the effective sample size (Aki et al., 2020). To assess the consistency assumption in the network, we also utilized functionalities within the netmeta package; for instance, by employing node-splitting analysis to assess differences between effect estimates from direct and indirect comparisons at specific treatment nodes (Dias et al., 2010). The network structure was visualized using a network plot to illustrate the direct relationship between different interventions. The final treatment effects were presented as pooled Hedges’ g and 95% credible intervals (CrIs). Furthermore, we calculated the Surface Under the Cumulative Ranking curve (SUCRA) values for each intervention to provide an exploratory summary of relative ranking probabilities compared with other interventions. Because treatment rankings may be unstable or potentially misleading when intervention nodes are sparsely informed or when effect estimates have wide CrIs, SUCRA values were interpreted together with pooled effect estimates, 95% CrIs, the number of contributing studies, direct evidence, and CINeMA certainty ratings (Nikolakopoulou et al., 2020; Salanti et al., 2011; Mbuagbaw et al., 2017). The results were presented using forest and ranking plots. Finally, we examined data from direct comparisons between each exercise intervention and the control group. For pairs in which the direct comparison included at least 10 studies, we used Egger’s linear regression to assess the degree of asymmetry between effect sizes and standard errors (Egger et al., 1997). We also calculated the S-value to quantify the extent of publication bias required to convert a statistically significant result into a non-significant one (Mathur and VanderWeele, 2020). Egger’s test was performed using the metafor R package, and the S-value was calculated using the PublicationBias package.

To evaluate the robustness of the Bayesian NMA findings, we conducted several sensitivity analyses. First, to assess the influence of SD imputation for change-from-baseline scores, we repeated the full Bayesian network meta-analysis under three assumed pre–post correlation coefficients: r = 0.2, r = 0.5, and r = 0.8. The change-score SDs and corresponding standard errors were recalculated under each assumed correlation coefficient before refitting the Bayesian NMA model. The model structure, priors, MCMC settings, and treatment coding were kept identical across these analyses. Second, to assess whether the main findings were driven by trials with high risk of bias, we conducted a risk-of-bias sensitivity analysis by excluding studies rated as high risk according to RoB 2.0 (Sterne et al., 2019). We also examined the feasibility of a more conservative low-risk-only analysis by excluding studies rated as either high risk or some concerns. Because only a small number of low-risk studies remained and several intervention nodes became sparsely represented, the low-risk-only analysis was treated as exploratory rather than definitive. The robustness of the findings was evaluated by comparing the direction, magnitude, 95% CrIs, and statistical interpretation of the intervention effects across the primary and sensitivity analyses.

Comparator heterogeneity was examined using three additional sensitivity analyses: excluding active comparator conditions, adding active comparator status as a model covariate, and separating the broad comparator node into four comparator-specific nodes: inactive control, usual care/standard treatment, active non-exercise control, and active physical control. Studies without a non-exercise control arm retained their original exercise–exercise comparison structure and were not treated as an additional comparator node. The plausibility of the transitivity assumption was assessed descriptively by summarizing potential effect modifiers across comparator categories. These analyses are reported in **Supplementary Tables S4**, **S5**, and the split-comparator network is shown in **Supplementary Figure 6**.

To investigate the relationship between exercise dosage and BDNF effect size, we conducted a dose-response meta-analysis. Specifically, we separately examined the potential non-linear relationships between Hedges’ g and duration of a particular exercise intervention in weeks, total weekly exercise dose, and exercise intensity (intensity_met, expressed in METs). These analyses were performed in the brms framework by introducing smooth spline functions for the dose-response variables in the model to fit the dose-effect curves (Bürkner, 2017). We explored the potential moderating effects of pre-defined covariates on the effectiveness of exercise interventions using a meta-regression analysis. The examined covariates included the mean age of the participants, percentage of females, and health status. These analyses were conducted using a multilevel Bayesian model by introducing the covariates and, if applicable, their interaction terms with the intervention type. The extent to which these factors contributed to the observed heterogeneity in effects was assessed by comparing the Bayesian R² values of models with and without these covariates (Gelman et al., 2019).

To aid statistical interpretation of the magnitude of BDNF changes, we calculated a 0.5-SD distribution-based interpretability threshold for the standardized effect size (Watt et al., 2021; Norman et al., 2003). The 0.5-SD approach can be used as a distribution-based benchmark for interpreting standardized changes; however, it should not be considered equivalent to a validated anchor-based minimal clinically important difference (MCID), because MCID/MID is intended to reflect clinically or patient-anchored meaningful change (Jaeschke et al., 1989; Revicki et al., 2008; de Vet et al., 2006). Therefore, in this review, the threshold was used only as a descriptive benchmark for comparing standardized effect sizes, rather than as evidence of clinically meaningful neuroprotection, cognitive preservation, or functional improvement. No validated anchor-based MCID for circulating BDNF levels in older adults was identified.

## 3 Results

### 3.1 Study selection

We initially obtained 1031 literature records through database searches. After removing 243 duplicate records, 788 articles were subjected to title and abstract screening, after which 715 articles were excluded. Subsequently, we conducted a detailed full-text assessment of 73 potentially eligible articles. Based on the pre-defined inclusion and exclusion criteria, 26 articles were ultimately excluded. Finally, 47 studies were included in this NMA (**Appendix A6**). The detailed literature screening process is illustrated in **Figure 1**.

### 3.2 Study characteristics

A total of 47 RCTs from 18 countries were included in this analysis, encompassing 1815 older participants. The detailed search strategy for identifying relevant studies is provided in **Appendix A1**. The control and intervention groups comprised 709 and 1106 participants, respectively. The mean age of the participants ranged from 55.8–92.3 years. The proportion of female participants varied across the studies, ranging from 0–100%. In most studies, the health status of the older participants allowed their engagement in exercise interventions, and most of these interventions were conducted under the supervision of professionals. The most common types of intervention were AE (15 study arms), RE (11 study arms), and ME (11 study arms). The total duration of the exercise interventions ranged from 1–48 weeks. For the detailed demographic characteristics of the included studies, refer to **Appendix A2**. The duration of a single exercise session ranged from 10–90 min. The prescribed exercise intensity ranged between 2–9 METs, resulting in a total weekly exercise dose of 210–1625 MET-min/week.

### 3.3 Risk of bias and evidence quality

The RoB 2.0 assessment results indicated that seven studies (14.9%) had a “low risk of bias,” 35 studies (74.5%) had “some concerns,” and the other five studies (10.6%) had a “high risk of bias.” **Appendix A3** presents a detailed summary of the risk of bias assessment. Regarding specific bias domains, “bias due to deviations from intended interventions” (with 76.6% of studies having some concerns) and “bias in the selection of the reported result” (with 55.3% of studies having some concerns and 4.3% being high-risk) were the main areas of concern. **Supplementary Figures S1**, **S2** illustrate the detailed risk of bias and the overall summary, respectively. The CINeMA assessment showed that although some exercise types demonstrated statistically reliable positive estimates, the overall confidence in evidence for various interventions was mostly “moderate” to “very low.” This was due to factors such as the common presence of some risk of bias in the original studies, limitations in assessing reporting bias for some comparisons, and significant overall heterogeneity in the network (**Table 1** provides the detailed CINeMA assessment results for each exercise intervention).

**Table 1:** Confidence ratings on the effects of exercise interventions on BDNF levels in older adults, assessed using the CINeMA framework.

Comparison (Intervention vs. Control)	NMA effect estimate (Hedges’ g [95% CrI])	Within-study bias	Reporting bias	Indirectness	Imprecision	Heterogeneity/Incoherence	Overall confidence in evidence	Rationale/Comments
Aquatic Exercise (AQE)	1.00 [0.44, 1.56]	Some concerns	Some concerns	No concerns	Major concerns	Some concerns	Low	Largest point estimate and favorable ranking, but evidence remains sparse:
								Imprecision: Very wide 95% CrI indicates unstable estimate.
								Within-study Bias: Based on only 2 studies (n=24); assumes average “Some concerns” from overall RoB.
								Reporting Bias: Few studies for direct comparison preclude full assessment; default “Some concerns”.
								Heterogeneity/Incoherence: Reflects overall network concerns.
Mind-Body Exercise (MBE)	0.61 [0.34, 0.89]	Some concerns	Some concerns	No concerns	No concerns	Some concerns	Moderate	Clear statistical effect; estimate exceeded the 0.5-SD distribution-based interpretability threshold:
								Imprecision: 95% CrI excludes 0, acceptable width.
								Within-study Bias: General “Some concerns” based on overall RoB.
								Reporting Bias: Assumed “Some concerns” due to potential limits in direct comparison assessment.
								Heterogeneity/Incoherence: Reflects overall network concerns.
Mixed Exercise (ME)	0.30 [0.13, 0.49]	Some concerns	Some concerns	No concerns	No concerns	Some concerns	Moderate	Clear statistical effect; point estimate below the 0.5-SD distribution-based interpretability threshold:
								Imprecision: 95% CrI excludes 0, acceptable width.
								Within-study Bias: General “Some concerns”.
								Reporting Bias: Assumed “Some concerns”.
								Heterogeneity/Incoherence: Reflects overall network concerns.
Aerobic Exercise (AE)	0.24 [0.08, 0.40]	Some concerns	Major concerns	Some concerns	No concerns	Some concerns	Low	Clear statistical effect; point estimate below the 0.5-SD distribution-based interpretability threshold, but: Indirectness: Comparator conditions in contributing studies included both inactive and active comparator conditions; sensitivity analyses excluding active comparators showed a robust direction but altered magnitude.
								Reporting Bias: Egger’s test for AE vs. Control direct comparison indicated significant asymmetry (p=0.005), thus “Major concerns”.
								Within-study Bias: “Some concerns”.
								Imprecision: 95% CrI excludes 0, acceptable width.
								Heterogeneity/Incoherence: Reflects overall network concerns.
Aerobic + Resistance (AE+RE)	0.58 [0.29, 0.87]	Some concerns	Some concerns	Some concerns	No concerns	Some concerns	Moderate	Clear statistical effect; estimate exceeded the 0.5-SD distribution-based interpretability threshold: Indirectness: Comparator conditions in contributing studies included both inactive and active comparator conditions; sensitivity analyses excluding active comparators showed a robust direction but altered magnitude.
								Imprecision: 95% CrI excludes 0, acceptable width.
								Within-study Bias: General “Some concerns”.
								Reporting Bias: Assumed “Some concerns”.
								Heterogeneity/Incoherence: Reflects overall network concerns.
Resistance Exercise (RE)	0.37 [0.20, 0.54]	Some concerns	Some concerns	No concerns	No concerns	Major concerns	Low	Clear statistical effect; point estimate below the 0.5-SD distribution-based interpretability threshold, but:
								Heterogeneity: Very high within-design heterogeneity for Control: RE comparisons (Q = 115.10) significantly impacts this domain; thus “Major concerns”.
								Within-study Bias: “Some concerns”.
								Reporting Bias: Assumed “Some concerns”.
								Imprecision: 95% CrI excludes 0, acceptable width.
HIIT	0.25 [-0.10, 0.60]	Some concerns	Some concerns	No concerns	Major concerns	Some concerns	Low	Uncertain statistical effect:
								Imprecision: 95% CrI includes 0; effect not statistically reliable.
								Within-study Bias: “Some concerns”.
								Reporting Bias: Assumed “Some concerns”.
								Heterogeneity/Incoherence: Reflects overall network concerns.
Coordination Exercise (CE)	0.11 [-0.23, 0.45]	Some concerns	Some concerns	No concerns	Major concerns	Some concerns	Very Low	Uncertain statistical effect; point estimate below the 0.5-SD distribution-based interpretability threshold:
								Imprecision: 95% CrI includes 0; effect not statistically reliable; point estimate below the 0.5-SD distribution-based interpretability threshold.
								Other domains assumed “Some concerns” as above.

This table summarizes the confidence ratings for the effects of various exercise interventions on BDNF levels in older adults, assessed using the CINeMA framework. SUCRA rankings and ranking-related comments should be interpreted as exploratory summaries and in conjunction with effect estimates, 95% CrIs, number of contributing studies, and CINeMA confidence ratings. Sparse nodes such as AQE and HIIT should not be interpreted as providing definitive evidence of superiority or effectiveness.

### 3.4 NMA

**Figure 2:**
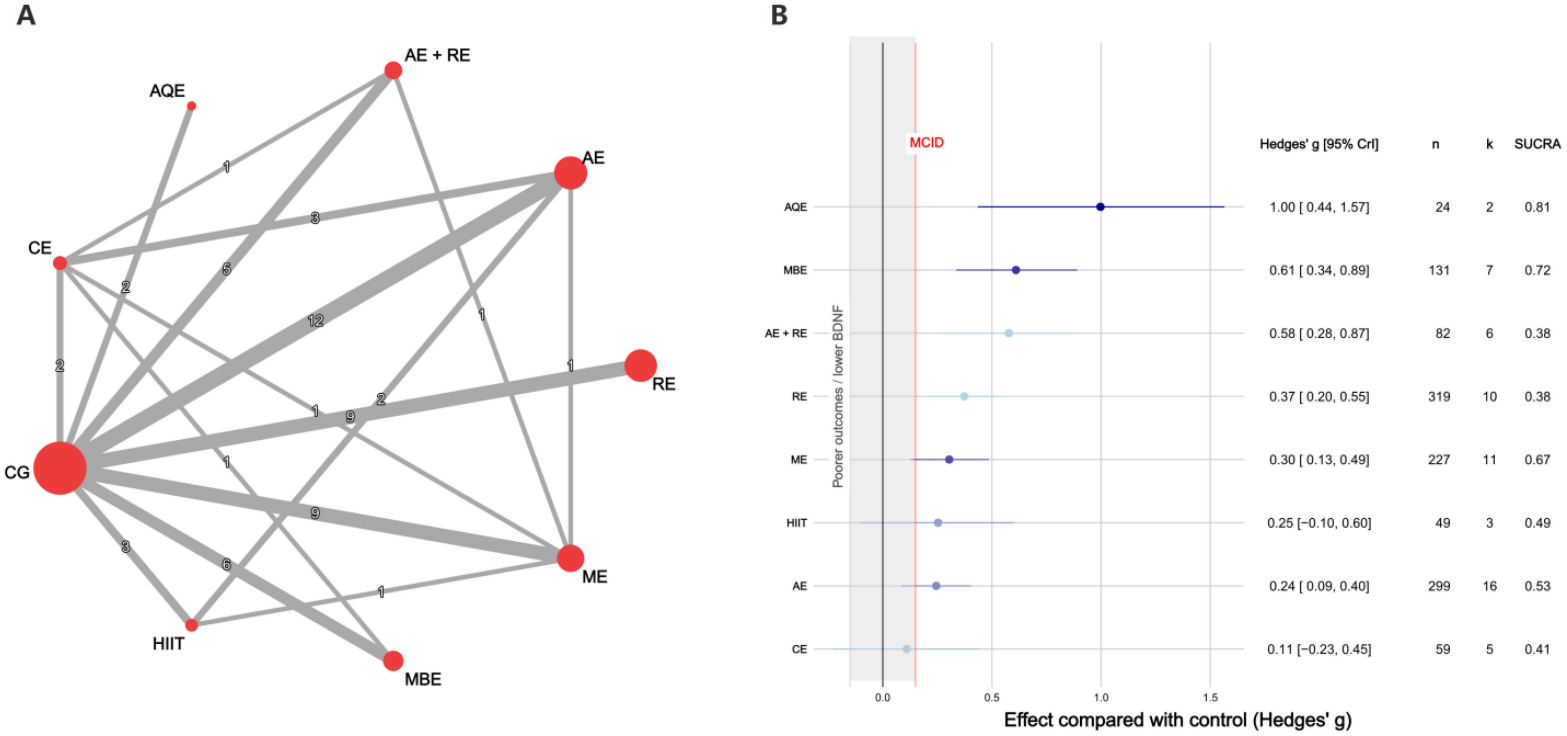
**(A)**. Network diagrams depicting the direct and indirect comparisons. This network diagram illustrates the direct and indirect comparisons for the network meta-analyses. The size of the nodes represents the number of participants in each intervention. The connections between the nodes represent a direct comparison of different exercise interventions, and their thickness indicates the amount of direct evidence. **(B)** Forest Plot of Study-Level Effects of Exercise on BDNF. This forest plot illustrates the effects of various exercise interventions on BDNF levels compared with the control group. SUCRA values are presented as exploratory ranking summaries. Higher SUCRA values indicate more favorable relative ranking probabilities; however, rankings should be interpreted together with effect estimates, 95% CrIs, evidence sparsity, and CINeMA confidence ratings, rather than as definitive evidence of treatment superiority. The figure includes continuous aerobic exercise (AE), coordinated exercise (CE), control group (CG), high-intensity interval training (HIIT), resistance exercise (RE), mind-body exercise (ME), aquatic exercise (AQE), and the combination of aerobic and resistance exercise (AE + RE).

A preliminary assessment revealed significant overall heterogeneity in the network (τ^2^ = 0.6365, I^2^ = 84%, 95% CI: 79.4–87.6%, Q total = 281.41, p <0.0001). Both within-design heterogeneity and between-design inconsistency were statistically significant, although the between-design inconsistency was not significant when more complex model assumptions were considered (**Figure 2A**, **Appendix A4**, **Supplementary Figure S3**). Compared with the control group, AQE showed the largest point estimate (SMD = 1.00, 95% CrI [0.44, 1.56]) and the highest SUCRA value (0.81) (**Figure 2B**). However, this finding should be interpreted cautiously rather than as definitive evidence of superiority, because it was based on only a small number of studies (k = 2, n = 24) and had a relatively wide 95% CrI. Several other exercise interventions also showed positive estimates. MBE (SMD = 0.61, 95% CrI [0.34, 0.89], SUCRA = 0.72) exceeded the 0.5-SD distribution-based interpretability threshold, whereas ME (SMD = 0.30, 95% CrI [0.13, 0.49], SUCRA = 0.67) showed a statistically reliable positive estimate but remained below this threshold (**Appendix A4**, **Supplementary Figure S4**). AE (SMD = 0.24, 95% CrI [0.08, 0.40], SUCRA = 0.53), AE + RE (SMD = 0.58, 95% CrI [0.29, 0.87], SUCRA = 0.38), and RE (SMD = 0.37, 95% CrI [0.20, 0.54], SUCRA = 0.38) also showed positive estimates; among these, AE + RE exceeded the 0.5-SD benchmark, whereas AE and RE remained below it. SUCRA values were interpreted as exploratory ranking summaries rather than as stand-alone evidence of treatment superiority. However, the SUCRA values for some interventions (such as AE + RE and RE) were relatively moderate, suggesting that their overall ranking might have been influenced by the magnitude of the effect size and/or the width of their CrIs. HIIT (SMD = 0.25, 95% CrI [−0.10, 0.60], SUCRA = 0.49) and CE (SMD = 0.11, 95% CrI [−0.23, 0.45], SUCRA = 0.41) showed favorable trends in point estimates, but their 95% CrIs included zero. Therefore, these effects remained statistically uncertain. In particular, HIIT should be interpreted as hypothesis-generating rather than as an established effective intervention, and further adequately powered trials are needed to confirm its effect on BDNF levels in older adults. According to the pre-specified criterion of requiring at least 10 studies, only the direct comparison of “AE vs. Control group” met the conditions for quantitative statistical analysis. The results of Egger’s test for this comparison showed significant asymmetry (p = 0.005), suggesting potential publication bias. However, the S-value could not be calculated using the available direct-comparison data and software implementation. All other direct comparisons between exercise interventions and the control group were not subjected to Egger’s test or S-value analysis because of the presence of fewer than 10 studies.

### 3.5 Sensitivity analyses

Sensitivity analyses using assumed pre–post correlation coefficients of r = 0.2, r = 0.5, and r = 0.8 showed that the overall pattern of findings remained stable. AE, AE + RE, AQE, MBE, ME, and RE consistently showed positive estimates with 95% CrIs excluding zero across all three models, whereas CE and HIIT remained uncertain, with 95% CrIs including zero. Across r = 0.2 to r = 0.8, the point estimates ranged from 0.20 to 0.27 for AE, 0.52 to 0.63 for AE + RE, 0.95 to 1.01 for AQE, 0.50 to 0.66 for MBE, 0.28 to 0.31 for ME, and 0.23 to 0.43 for RE. These results suggest that the main network estimates were not materially altered by plausible changes in the assumed pre–post correlation coefficient. The complete results are provided in **Supplementary Table S1**.

After excluding the five studies rated as high risk of bias, the main findings were generally preserved. AE, AE + RE, AQE, MBE, ME, and RE remained associated with positive estimates, whereas CE and HIIT remained uncertain. The estimates for AE, AQE, MBE, and ME were highly similar between the primary and high-risk-exclusion analyses, while AE + RE remained positive with a slightly smaller estimate. The estimate for RE increased after excluding high-risk studies, indicating that the magnitude of this comparison should still be interpreted cautiously. The feasibility of a more conservative low-risk-only analysis was also examined; however, because only seven low-risk studies remained and several intervention nodes became too sparse for reliable comparative inference, this analysis was regarded as exploratory rather than definitive. The complete high-risk-exclusion sensitivity results are provided in **Supplementary Table S2**.

Comparator/control conditions were classified as inactive control (25 studies), usual care/standard treatment (4 studies), active non-exercise control (8 studies), active physical control (6 studies), or no non-exercise control arm (4 studies; **Supplementary Table S3**). The last category represented exercise–exercise comparisons and was not treated as an additional comparator node. Comparator-handling sensitivity analyses revealed an important pattern: after excluding active comparator conditions, the effect estimates for several exercise interventions became larger. In particular, the AE estimate increased from 0.245 (95% CrI 0.086 to 0.403) to 0.369 (95% CrI 0.129 to 0.614), the ME estimate increased from 0.305 (95% CrI 0.135 to 0.475) to 0.526 (95% CrI 0.306 to 0.746), and the HIIT estimate changed from statistically uncertain in the primary analysis (0.253, 95% CrI -0.097 to 0.598) to positive after excluding active comparator conditions (0.551, 95% CrI 0.082 to 1.023). The HIIT finding should still be interpreted cautiously because the evidence remained sparse and the CrI was wide. In the separated-comparator-node analysis, active non-exercise comparator conditions and active physical comparator conditions showed positive trends relative to inactive control (0.243, 95% CrI -0.009 to 0.499; and 0.187, 95% CrI -0.075 to 0.454, respectively), whereas usual care/standard treatment showed no apparent BDNF response (-0.088, 95% CrI -0.326 to 0.151). These findings suggest that combining active and inactive comparator conditions into a single broad comparator node may have attenuated the apparent effects of some exercise interventions relative to inactive control. When active comparator status was added as a covariate, the overall coefficient for active comparator status was small and uncertain (0.027, 95% CrI -0.187 to 0.246), indicating no clear systematic shift across all interventions; however, individual intervention estimates in the covariate model showed some variation, such as AE + RE (0.793, 95% CrI 0.471 to 1.116) compared with the primary analysis (0.579, 95% CrI 0.276 to 0.877). Full results are provided in **Supplementary Table S4**; potential effect modifiers across comparator categories are summarized in **Supplementary Table S5**, and the split-comparator network is shown in **Supplementary Figure S6**.

### 3.6 Dose-response analysis

**Figure 3:**
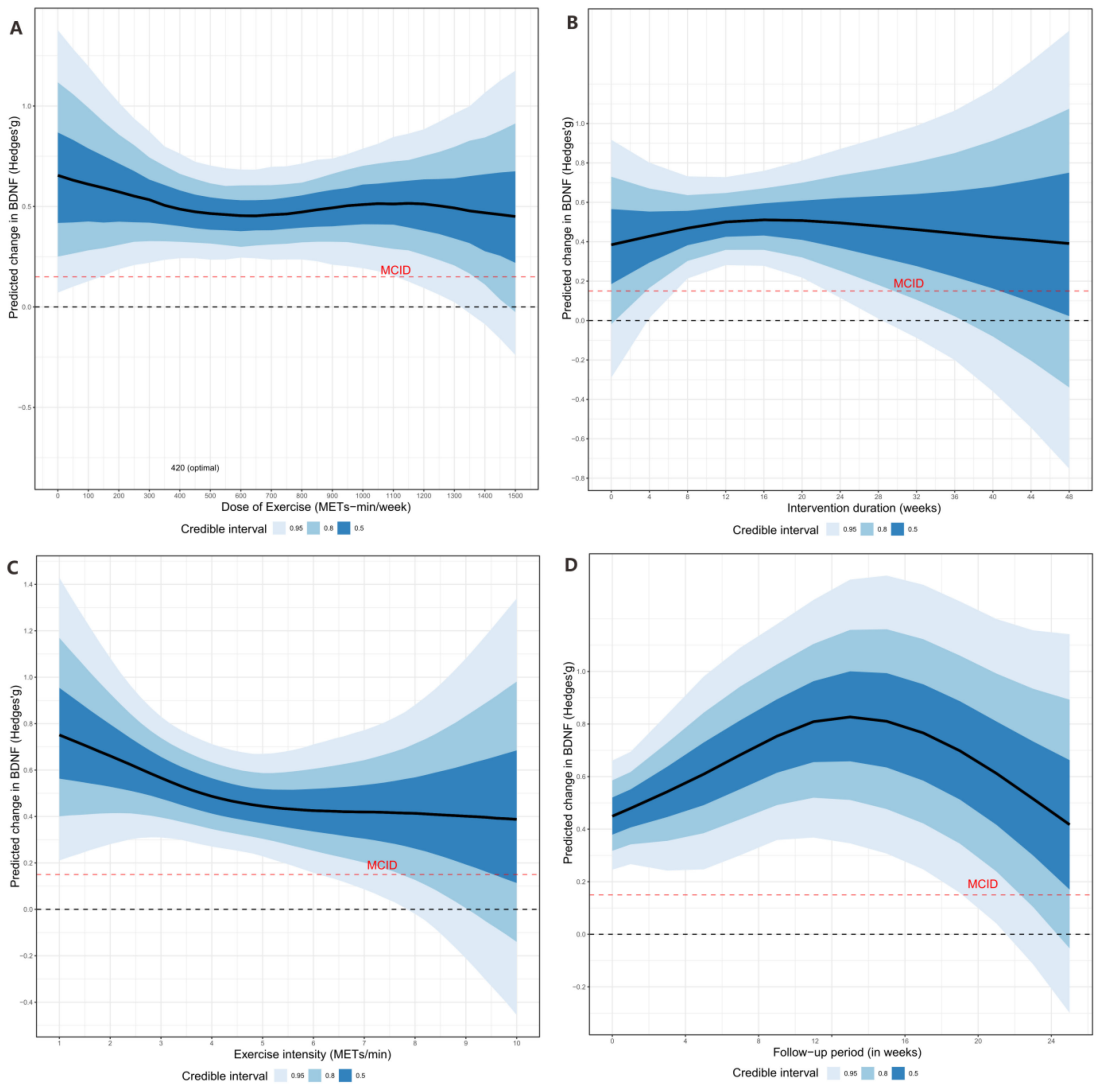
**(A)**. Dose-response of total exercise dose and BDNF effects. This plot shows the predicted change in BDNF levels as a function of total weekly exercise dose (MET-minutes/week). The curve demonstrates a non-linear relationship, with effect sizes maintained at a high level across various dosage levels. The shaded area represents the credible interval, illustrating the uncertainty around the point estimates. The red dashed line indicates the 0.5-SD distribution-based interpretability threshold. **(B)** Dose-Response of Exercise Duration and BDNF Effects. This plot illustrates the predicted change in BDNF levels in response to the duration of exercise interventions (in weeks). The effect size increases initially, peaking at 8–20 weeks, and stabilizes thereafter. The credible intervals reflect the uncertainty around the predicted effect at different time points, with the 0.5-SD distribution-based interpretability threshold indicated by the red dashed line. **(C)** Dose-response of exercise intensity and BDNF effects. This plot examines the relationship between exercise intensity (METs) and the predicted change in BDNF levels. The figure shows that lower intensities result in higher BDNF effect sizes, which gradually decrease as intensity increases. The credible interval and the 0.5-SD distribution-based interpretability threshold are represented, showing that excessive intensity does not necessarily yield better results. **(D)** Dose-response of follow-up duration and BDNF effects. This plot depicts the predicted change in BDNF levels during the follow-up period after the exercise intervention. The effect size peaks at 12–16 weeks and remains stable at relatively high levels for up to 24 weeks, suggesting sustained benefits even after exercise cessation. The credible interval and the 0.5-SD distribution-based interpretability threshold are indicated, providing statistical insight into the durability of the exercise effects. This threshold was used as a statistical benchmark rather than as a validated clinical MCID.

All dose-response analyses indicated that the predicted effect sizes, or the majority of their CrIs, generally remained above the 0.5-SD distribution-based interpretability threshold. Specifically, the relationship between the total weekly exercise dose (MET-min/week) and BDNF level was non-linear, with maintained effect sizes across various dosage levels (**Figure 3A**). For exercise intensity (METs), the results suggested a higher BDNF effect size at lower intensities, which decreased slightly before stabilizing as the intensity increased (**Figure 3C**), indicating that a higher intensity does not necessarily yield better results. Regarding the duration of intervention in weeks, the BDNF effect size slightly increased initially, peaking at approximately 8–20 weeks. Although a very slow declining trend was observed thereafter, the predicted effect remained above the distribution-based interpretability threshold for up to 48 weeks (**Figure 3B**). Notably, while examining the influence of follow-up duration in weeks after the intervention, the BDNF effect did not immediately cease after the exercise was stopped; instead, it peaked at 12–16 weeks of follow-up and then gradually declined, remaining at a relatively high level even at 24 weeks (**Figure 3D**), suggesting sustained and potentially delayed effects of exercise (**Appendix A5**).

### 3.7 Meta-regression

**Figure 4:**
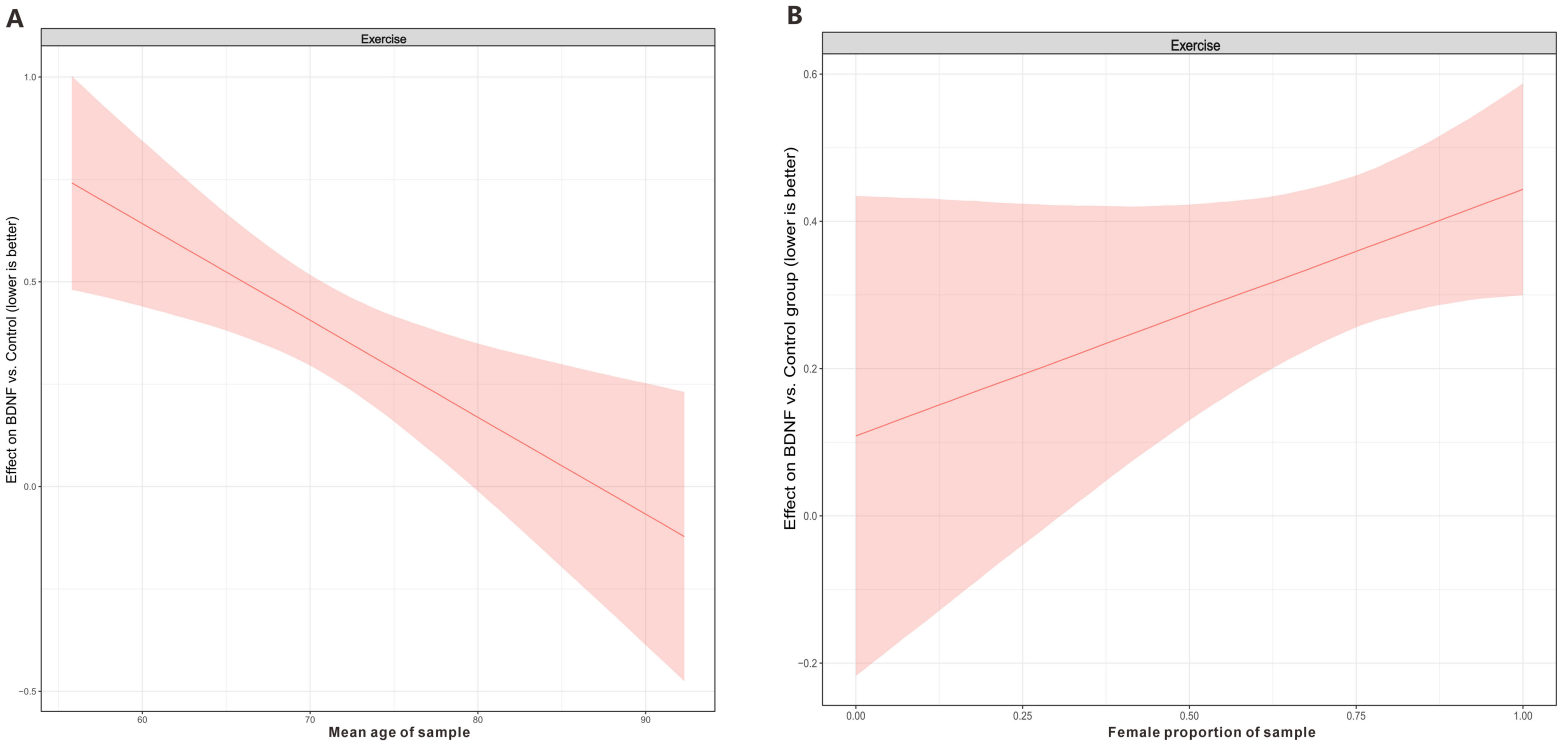
**(A)**. A Effect of Age on BDNF Effects. This plot illustrates the relationship between the mean age of the sample and the estimated exercise effect on BDNF levels, with lower predicted effect sizes indicating smaller exercise-related increases in BDNF. The figure shows a negative trend, where older age correlates with a lower estimated effect on BDNF, suggesting that the impact of exercise may decrease as age increases. The shaded area around the line represents the credible interval, indicating the uncertainty of the effect at different ages. **(B)** Effect of Percent Female on BDNF Effects. This plot displays the relationship between the percentage of female participants in the study and the estimated exercise effect on BDNF levels, with higher predicted effect sizes indicating stronger exercise-related increases in BDNF. The positive trend suggests that a higher proportion of female participants is associated with a stronger estimated effect of exercise on BDNF. The shaded area indicates the credible interval, providing a range of uncertainty for the relationship.

The meta-regression results indicated a negative correlation between the average age of participants and the exercise effect (**Figure 4A**): the beneficial effect of exercise on BDNF level diminished as the average sample age increased. In very old individuals (aged approximately 90 years), the uncertainty of the effect increased (the 95% CrI included 0). Regarding the percentage of female participants (**Figure 4B**), the analysis suggested a potentially positive moderating trend, implying that the exercise effect appeared better when the proportion of female participants was higher. However, in studies with a low proportion of females, this trend was not evident, and the 95% CrI included 0; the evidence for this trend was relatively clear only when the proportion of females was high.

## 4 Discussion

### 4.1 Main finding

This NMA systematically evaluated the effects of different exercise types on BDNF level in 1815 older adults across 47 RCTs. Compared with the control groups, several exercise interventions, including AQE, MBE, ME, AE, AE + RE, and RE, showed statistically reliable positive estimates for BDNF levels. Among these, AQE, MBE, and AE + RE exceeded the 0.5-SD distribution-based interpretability threshold, whereas ME, AE, and RE showed positive but smaller standardized effects. However, this threshold should be interpreted only as a statistical benchmark for effect magnitude and not as a validated clinical MCID for circulating BDNF (Watt et al., 2021; Norman et al., 2003; Jaeschke et al., 1989; Revicki et al., 2008; de Vet et al., 2006). Among these interventions, AQE exhibited the largest point estimate and the most favorable SUCRA value; however, this finding should be interpreted cautiously because it was informed by only a small number of studies and had limited precision. HIIT showed a favorable point estimate but remained statistically uncertain; therefore, it should be regarded as hypothesis-generating. Dose-response analysis revealed complex non-linear relationships between exercise parameters (total weekly dose, intensity, intervention duration, and follow-up duration) and BDNF effects. Most predicted effects exceeded the 0.5-SD distribution-based interpretability threshold, and the positive impact of exercise might show a delayed peak even after cessation of the intervention. However, whether these biomarker changes translate into measurable cognitive, neurological, or functional benefits remains uncertain. In addition, an optimal dose window could not be identified because of limited data from studies with extreme dosages. The meta-regression analysis showed that the average age of the participants negatively moderated the exercise effect, whereas a higher percentage of female participants might be associated with better outcomes, although the latter requires more evidence. Despite these findings, the overall risk of bias in the original studies, significant overall heterogeneity within the network, and potential publication bias in some comparisons resulted in the confidence in evidence (assessed by CINeMA) for various interventions being mostly “moderate” to “very low.”

### 4.2 Comparison with previous studies

Previous studies have focused on specific exercise types or conducted limited direct comparisons. For example, Marinus et al. (2019) reported positive effects of RE and combined training on BDNF levels in older adults, whereas low-to-moderate-intensity AE did not show a clear effect. The present study also supported positive estimates for RE and combined exercise, and in our network, AE showed a statistically reliable positive estimate, although its point estimate remained below the 0.5-SD distribution-based interpretability threshold. Nevertheless, this benchmark should not be interpreted as direct evidence of clinically meaningful cognitive or functional improvement (Watt et al., 2021). This could be because our study included a broader range of studies, or because the multilevel Bayesian model provided superior handling of heterogeneity and differed in the method of effect size estimation. Setayesh and Mohammad Rahimi (2023) also reported a positive effect of RE on BDNF levels in older adults, which is consistent with our results. Furthermore, recent meta-analyses focusing on older or middle-aged and older adults reported positive effects of exercise training on BDNF levels, although the magnitude of response may vary by exercise modality, health status, weekly exercise duration, blood sampling timing, and sample source. This is broadly consistent with the positive estimates observed for several exercise categories in our study (Gholami et al., 2025; Marinus et al., 2019; Li et al., 2025).

Compared with the findings of Dinoff et al. (2016) in a population with a broader age range (mean age 42.2 years, including older adults), which found AE effective but not RE, the present study and several other studies focusing primarily on older adults showed the positive role of RE on BDNF level in this population. This discrepancy may be due to age-related physiological changes, leading to potentially different BDNF response patterns to various exercise types in older adults compared to that in younger or middle-aged individuals (Walsh et al., 2020). Older adults commonly face muscle decline (sarcopenia), and RE, which stimulates muscle growth and improves metabolic function, can upregulate BDNF level through unique “muscle-brain crosstalk” pathways (Gholami et al., 2025; Cruz-Jentoft et al., 2019; Rai and Demontis, 2022). For example, muscles release various myokines such as irisin during contraction. These factors are believed to cross the blood-brain barrier and promote BDNF expression in the hippocampus (Wrann, 2015; Wrann et al., 2013; Vints et al., 2023). Furthermore, RE can improve insulin sensitivity, and insulin signaling pathways are closely related to BDNF expression and function (Jiahao et al., 2021; Grillo et al., 2015; Spinelli et al., 2019).

In the present study, MBE, such as Tai Chi and Yoga, also showed a positive estimate with a 95% CrI excluding zero. Similarly, Gan et al. (2025) reported that traditional Chinese exercises were associated with increased BDNF levels in middle-aged and older adults, particularly in individuals who were cognitively normal. MBE not only incorporates low-to-moderate intensity physical activity but also emphasizes the integration of breath regulation, focused meditation, and proprioception (Cahn et al., 2017). These elements exert their effects through multiple mechanisms, such as regulating autonomic nervous system function by reducing sympathetic nervous system excitability and increasing parasympathetic tone (Kuppusamy et al., 2020; Motivala et al., 2006); alleviating psychological stress levels, thereby diminishing the inhibitory effect of stress hormones, such as cortisol, on BDNF (Koncz et al., 2021); and indirectly promoting BDNF production and release by improving inflammatory status (Bower and Irwin, 2016).

AQE showed the largest point estimate and a favorable ranking profile (**Appendix A4**, **Supplementary Figure S5**). Buoyant properties of the water environment reduce the load on joints during exercise in older adults, potentially enabling them to perform movements with a greater range, for a longer duration, or with a higher probability of completion (Kutzner et al., 2017). Concurrently, water pressure and temperature might also exert unique stimuli on the circulatory and nervous systems, thereby influencing BDNF levels (Kojima et al., 2018). However, this interpretation is primarily based on a small number of studies (k = 2), and AQE should therefore be regarded as a promising but preliminary exercise modality requiring confirmation in larger, high-quality trials.

The effectiveness of ME or multicomponent exercise was also supported by the results of this study, which complements the conclusion by Wang et al. that research on the impact of multicomponent exercise on BDNF levels in older adults with MCI or dementia is limited, but shows positive trends (Wang et al., 2020). ME demonstrates favorable effects because it integrates the benefits of multiple exercise modalities. For instance, aerobic components improve cerebral blood flow and overall metabolism (Dhahbi et al., 2025; Kleinloog et al., 2019) and resistance components activate myokines (Rai and Demontis, 2022; Gao et al., 2024), whereas balance and CE increase neural recruitment and improve proprioceptive input (Kubica et al., 2019; Rogge et al., 2017). These factors collectively promote neuroplasticity and increase BDNF levels.

In summary, the differences in the effects of various exercise types on BDNF levels may stem from the distinct physiological pathways that they activate, their differing metabolic demands, and their varying emphasis on regulating neuroendocrine and inflammatory states. The results of this NMA underscore that no single exercise is a “one-size-fits-all” type; each form of exercise possesses a unique value and potential mechanism. This provides a broader perspective and wide array of options for developing individualized exercise prescriptions that cater to the diverse needs and preferences of older adults.

Regarding the dose-response analysis, although positive BDNF responses were observed across various doses, an optimal dose window could not be clearly defined because of the scarcity of original research that assessed extreme dosages (either very low or very high). For exercise intensity, the results suggest that a higher intensity is not necessarily better, as the predicted BDNF response remained favorable even at low to moderate intensities. The analysis of intervention duration indicated that the BDNF elevation might reach a plateau or peak at approximately 8–20 weeks and then be largely maintained. Interestingly, the positive impact of the exercise intervention may continue to increase or peak during the follow-up period even after cessation (approximately 12–16 weeks), highlighting the sustained and potentially delayed biological effects of exercise. These findings emphasize that when formulating exercise prescriptions, one should seek an optimal combination and balance of various dosage parameters rather than solely pursuing the maximization of a single parameter, and consider the dynamic changes and long-term impact of exercise. With advancing age, the magnitude of exercise-induced BDNF increments may diminish. This could be related to a decline in neuroplastic reserves and changes in the physiological adaptation capacity in very old individuals, suggesting that exercise programs for older adults of different age groups require individualized adjustments.

In this study, when the proportion of female participants was higher, the effect of exercise was more pronounced. However, this trend was less clear in studies with a low female representation, indicating that sex factors may influence the effect of exercise on BDNF levels. The specific mechanisms and interactions warrant future in-depth research to provide targeted exercise recommendations for older adults of different sexes.

### 4.3 Practical implications

The results of this study may have implications for exercise prescription and public health practice. Various forms of exercise, including AQE, MBE, and ME, may increase BDNF levels in older adults and provide evidence to inform non-pharmacological intervention strategies. Among these, MBE and ME may be considered because they showed relatively consistent effects, aiding in the scientific recommendation of exercise types based on individual circumstances (Gan et al., 2025; Kim and Kim, 2018). Furthermore, extreme exercise dosage (such as intensity and duration) is not necessarily better; moderate-intensity interventions lasting 8–20 weeks can provide favorable effects, and the benefits can remain sustained or even increase after the intervention period. This provides crucial information for optimizing exercise prescriptions and improving engagement and long-term adherence among older adults (Rahmi et al., 2022; Yuan et al., 2024). Lastly, considering that individual differences such as age and sex may influence exercise outcomes, this study underscores the importance of promoting individualized exercise interventions for different groups of older adults, with the aim of supporting potential BDNF-related benefits and broader health outcomes (Vaughan et al., 2014).

### 4.4 Strengths and limitations

The main strengths of this study are as follows. First, it employed an advanced multilevel Bayesian NMA methodology, which allows simultaneous direct and indirect comparisons of multiple exercise types and provides a probabilistic ranking, thereby overcoming the limitations of traditional pairwise meta-analyses. Second, this model can assess between-study heterogeneity better and allows more robust estimations in the presence of potential data dependencies (such as in multi-arm studies), while also providing a probabilistic interpretation of effect sizes (95% CrIs). Third, this study comprehensively and systematically included various exercise intervention types commonly advised to older populations, and specifically conducted an in-depth analysis targeting this demographic. Fourth, we further explored the dose-response relationships between key exercise parameters and BDNF effects and analyzed important moderating factors, such as age and sex, providing valuable cues for the individualization and precision of exercise prescriptions.

However, this study has some limitations. First, although we attempted to conduct a comprehensive and meticulous literature search, the potential for selection and publication biases cannot be ruled out. Second, the risk-of-bias assessment showed that most included studies were rated as having some concerns, which directly affected the overall confidence in the evidence. Although the sensitivity analysis excluding studies rated as high risk of bias showed that the main findings were generally preserved, only seven studies were rated as low risk. Therefore, a low-risk-only analysis was not considered suitable for definitive network inference because several intervention nodes would become sparsely represented. Residual within-study bias cannot be fully excluded and was therefore reflected in the CINeMA confidence ratings. Third, because some included studies did not directly report SDs of change-from-baseline scores, these SDs had to be imputed using assumed pre–post correlations. Although sensitivity analyses using r = 0.2, r = 0.5, and r = 0.8 showed that the main findings were generally robust, this imputation procedure remains a potential source of methodological uncertainty.

Fourth, the interpretation of the 0.5-SD threshold should be considered with caution. No validated anchor-based MCID for circulating BDNF levels in older adults was identified. Although peripheral BDNF is biologically relevant and has been associated with cognitive function and mild cognitive impairment in older adults (Shimada et al., 2014), changes in circulating BDNF cannot be assumed to directly translate into measurable neuroprotection, cognitive preservation, or functional improvement. Therefore, the 0.5-SD threshold used in this review should be interpreted only as a distribution-based statistical benchmark for standardized effect magnitude (Watt et al., 2021; Norman et al., 2003), rather than as a validated anchor-based MCID, which should be grounded in clinically or patient-anchored meaningful change (Jaeschke et al., 1989; Revicki et al., 2008; de Vet et al., 2006). Future trials should jointly assess BDNF and validated cognitive, neurological, or functional outcomes to determine whether exercise-induced BDNF changes have clinically meaningful implications. In addition, BDNF quantification varied across studies in terms of biological sample source, assay procedures, reporting units, and timing of assessment. Although the use of Hedges’ g placed effects on a standardized scale, assay-related and sample-source heterogeneity could not be fully removed or formally explored because reporting details were limited in some trials.

Fifth, comparator heterogeneity remains an important methodological limitation. Although the primary analysis retained a pragmatic broad-comparator reference node to preserve network connectivity, comparator conditions in exercise trials may differ in contact, expectancy effects, behavioral activation, and physical activity content. We therefore reclassified comparator/control conditions, performed comparator-handling sensitivity analyses, summarized potential effect modifiers across comparator categories, and provided a split-comparator network plot. The main findings were generally preserved; however, residual comparator-related and transitivity-related uncertainty cannot be fully excluded, particularly for sparsely represented comparator categories and intervention nodes. Studies without a non-exercise control arm were handled as exercise–exercise comparisons and were not treated as a fifth comparator node.

Sixth, significant overall heterogeneity remained in the network, and the number of included studies was relatively small for certain exercise types, particularly AQE and HIIT. Therefore, AQE, HIIT, and SUCRA rankings should be interpreted cautiously, and the findings should be regarded as biomarker-level evidence rather than definitive clinical recommendations.

## 5 Conclusion

The results of this multilevel Bayesian NMA suggest that several exercise modalities, including AQE, MBE, ME, AE, AE + RE, and RE, may increase BDNF levels in older adults. Comparator-handling sensitivity analyses generally supported the main pattern of findings, although residual comparator-related and transitivity-related uncertainty should be considered when interpreting the results. Furthermore, the point estimates for AQE, MBE, and AE + RE exceeded the 0.5-SD distribution-based interpretability threshold, whereas other statistically positive interventions showed smaller standardized effects. However, because no validated anchor-based MCID for circulating BDNF in older adults is currently available, the clinical implications of these biomarker changes should be interpreted cautiously. AQE showed the largest point estimate but should be interpreted cautiously because of sparse evidence, whereas HIIT remains hypothesis-generating because its primary-analysis estimate was statistically uncertain, although the active-comparator-exclusion sensitivity analysis yielded a positive estimate. Complex non-linear relationships exist among exercise dosage parameters, duration of effects after intervention cessation, and BDNF levels. Simultaneously, the mean age and possibly sex proportion of participants are potential moderating factors influencing exercise effectiveness. These findings may inform individualized exercise prescriptions and future research on BDNF-related exercise responses. Future research should focus on more rigorously designed, adequately powered, and high-quality RCTs. Particularly, exercise types for which evidence is currently insufficient, such as AQE, should be researched. Direct comparisons between different exercise programs should be strengthened to clarify appropriate exercise prescriptions for older adults with varying characteristics and underlying neurobiological mechanisms. The ultimate aim is to effectively maintain and improve cognitive function, overall health, and well-being in older adults using exercise intervention strategies.
