# Supplementary material for: Optimizing exercise modalities to enhance brain-derived neurotrophic factor levels in older adults: a Bayesian network and dose-response meta-analysis
Source: Front Physiol. 2026 Jul 15;17:1777058. doi: 10.3389/fphys.2026.1777058 (PMC13414880; doi:10.3389/fphys.2026.1777058)
Supplement: Supplementary file 1 [file DataSheet1.docx]

**Optimizing Exercise Modalities to Enhance Brain-Derived Neurotrophic Factor Levels in Older Adults: A Bayesian Network and Dose-Response Meta-Analysis**

**Supplemental files**

**Contents**

[**Appendix 1: Search strategy 1**](#_Toc230306769)

[**Appendix 2: Demographic characteristics of included studies 3**](#_Toc230306770)

[**Appendix 3: Within-study risk of bias 9**](#_Toc230306771)

[**Appendix 4: Bayesian Network Meta-Analysis of Exercise and BDNF in Older Adults 10**](#_Toc230306772)

[**4.1 Posterior R² distributions of predictor models** 10](#_Toc230306773)

[**4.2 Subgroup analysis of exercise effects on BDNF in healthy and unhealthy older adults** 11](#_Toc230306774)

[**4.3 Forest plot of study-level effects of exercise on BDNF** 12](#_Toc230306775)

[**4.4 Sensitivity analyses and transitivity assessment** 13](#_Toc230306776)

[4.4.1 Supplementary Table S1. Sensitivity analyses using different assumed pre–post correlation coefficients for change-score SD imputation. 13](#_Toc230306777)

[4.4.2 Supplementary Table S2. Sensitivity analysis excluding studies rated as high risk of bias. 14](#_Toc230306778)

[4.4.3 Supplementary Table S3. Comparator/control classification across included studies 15](#_Toc230306779)

[4.4.4 Supplementary Table S4. Comparator-handling sensitivity analyses 25](#_Toc230306780)

[4.4.5 Supplementary Table S5. Assessment of potential effect modifiers across comparator categories. 27](#_Toc230306781)

[4.4.6 Supplementary Figure S6. Split-comparator network plot. 28](#_Toc230306782)

[**Appendix 5: Predicted responses 29**](#_Toc230306783)

[**Appendix 6: Included randomized controlled trials contributing data to the meta-analysis 31**](#_Toc230306784)

# Appendix 1: Search strategy

1. PubMed

| **Search number** | **Query** | **Results** |
| --- | --- | --- |
| 1 | Exercise[MeSH Terms] OR Physical Activity[Title/Abstract] OR Activities, Physical[Title/Abstract] OR Activity, Physical[Title/Abstract] OR Physical Activities[Title/Abstract] OR Exercise, Physical[Title/Abstract] OR Exercises, Physical[Title/Abstract] OR Physical Exercise[Title/Abstract] OR Physical Exercises[Title/Abstract] OR Exercise, Acute[Title/Abstract] OR Exercises, Acute[Title/Abstract] OR Exercise, Isometric[Title/Abstract] OR Exercises, Isometric[Title/Abstract] OR Isometric Exercises[Title/Abstract] OR Isometric Exercise[Title/Abstract] OR Exercise, Aerobic[Title/Abstract] OR Aerobic Exercise[Title/Abstract] OR Aerobic Exercises[Title/Abstract] OR Exercises, Aerobic[Title/Abstract] OR Exercise Training[Title/Abstract] OR Exercise Trainings[Title/Abstract] OR Training, Exercise[Title/Abstract] OR Trainings, Exercise[Title/Abstract] OR Tai chi[Title/Abstract] OR Yoga[Title/Abstract] OR Pilates[Title/Abstract] OR Aquatic exercise[Title/Abstract] OR Qi gong[Title/Abstract] OR Wu qin xi[Title/Abstract] OR Stretch[Title/Abstract] OR Strength[Title/Abstract] | 886453 |
| 2 | “Brain‑Derived Neurotrophic Factor”[Mesh] OR Brain‑Derived Neurotrophic Factor[Title/Abstract] OR Factor, Brain‑Derived[Title/Abstract] OR Neurotrophic Factor, Brain‑Derived[Title/Abstract] OR BDNF[Title/Abstract] | 35465 |
| 3 | “Aged”[Mesh] OR elderly[Title/Abstract] OR aged[Title/Abstract] | 4332089 |
| 4 | Randomized controlled trial[Title/Abstract] OR Randomized[Title/Abstract] OR Placebo[Title/Abstract] | 890343 |
| 5 | #1 AND #2 AND #3 AND #4 | 110 |

1. Embase

| **Search number** | **Query** | **Results** |
| --- | --- | --- |
| 1 | 'exercise'/exp OR 'physical activity':ab,ti OR 'activities, physical':ab,ti OR 'activity, physical':ab,ti OR 'physical activities':ab,ti OR 'exercise, physical':ab,ti OR 'exercises, physical':ab,ti OR 'physical exercise':ab,ti OR 'physical exercises':ab,ti OR 'exercise, acute':ab,ti OR 'exercises, acute':ab,ti OR 'exercise, isometric':ab,ti OR 'exercises, isometric':ab,ti OR 'isometric exercises':ab,ti OR 'isometric exercise':ab,ti OR 'exercise, aerobic':ab,ti OR 'aerobic exercise':ab,ti OR 'aerobic exercises':ab,ti OR 'exercises, aerobic':ab,ti OR 'exercise training':ab,ti OR 'exercise trainings':ab,ti OR 'training, exercise':ab,ti OR 'trainings, exercise':ab,ti OR 'tai chi':ab,ti OR yoga:ab,ti OR pilates:ab,ti OR 'aquatic exercise':ab,ti OR 'qi gong':ab,ti OR 'wu qin xi':ab,ti OR stretch:ab,ti OR strength:ab,ti | 116334 |
| 2 | 'brain derived neurotrophic factor'/exp OR 'brain derived neurotrophic factor':ab,ti OR BDNF:ab,ti OR 'neurotrophic factor':ab,ti OR neurotrophin:ab,ti | 75159 |
| 3 | 'aged'/exp OR elderly:ab,ti OR aged:ab,ti | 5133763 |
| 4 | 'randomized controlled trial':ab,ti OR randomized:ab,ti OR placebo:ab,ti | 1315210 |
| 5 | #1 AND #2 AND #3 AND #4 | 189 |

1. Cochrane Library

| **Search number** | **Query** | **Results** |
| --- | --- | --- |
| 1 | "physical activity":ti,ab,kw OR "activities, physical":ti,ab,kw OR "activity, physical":ti,ab,kw OR "physical activities":ti,ab,kw OR "exercise, physical":ti,ab,kw OR "exercises, physical":ti,ab,kw OR "physical exercise":ti,ab,kw OR "physical exercises":ti,ab,kw OR "exercise, acute":ti,ab,kw OR "exercises, acute":ti,ab,kw OR "exercise, isometric":ti,ab,kw OR "exercises, isometric":ti,ab,kw OR "isometric exercises":ti,ab,kw OR "isometric exercise":ti,ab,kw OR "exercise, aerobic":ti,ab,kw OR "aerobic exercise":ti,ab,kw OR "aerobic exercises":ti,ab,kw OR "exercises, aerobic":ti,ab,kw OR "exercise training":ti,ab,kw OR "exercise trainings":ti,ab,kw OR "training, exercise":ti,ab,kw OR "trainings, exercise":ti,ab,kw OR "tai chi":ti,ab,kw OR "yoga":ti,ab,kw OR "pilates":ti,ab,kw OR "aquatic exercise":ti,ab,kw OR "qi gong":ti,ab,kw OR "wu qin xi":ti,ab,kw OR "stretch":ti,ab,kw OR "strength":ti,ab,kw | 138934 |
| 2 | "brain derived neurotrophic factor":ti,ab,kw OR BDNF:ti,ab,kw OR "neurotrophic factor":ti,ab,kw OR neurotrophin:ti,ab,kw | 2213 |
| 3 | elderly:ti,ab,kw OR aged:ti,ab,kw OR "older adult*":ti,ab,kw OR "older people":ti,ab,kw | 30811 |
| 3 | "randomized controlled trial":ti,ab,kw OR randomized:ti,ab,kw OR placebo:ti,ab,kw | 721619 |
| 4 | #1 AND #2 AND #3 AND #4 | 494 |

1. Web of Science

| **Search number** | **Query** | **Results** |
| --- | --- | --- |
| 1 | TS=(exercise OR "physical activity" OR "aerobic exercise" OR "resistance training" OR "tai chi" OR yoga OR pilates OR stretch OR strength OR walking OR cycling OR running) | 5771830 |
| 2 | TS=("brain derived neurotrophic factor" OR BDNF) | 41943 |
| 3 | TS=(elderly OR aged) | 5162170 |
| 4 | TS=("randomized controlled trial" OR randomized OR placebo) | 1386054 |
| 5 | #1 AND #2 AND #3 AND #4 | 239 |

# Appendix 2: Demographic characteristics of included studies

| **Country** | **n** | **% Female** | **Mean Age (SD/Range)** | **Session_duration(min)** | **Health Status** | **Supervise** | **Treatment** | **Duration (weeks)** | **Intensity prescribed (METs)** | **Exercise dose (METs/week)** | **Timepoints available** | **Outcomes measured** |
| --- | --- | --- | --- | --- | --- | --- | --- | --- | --- | --- | --- | --- |
| ArsalanDamirchi2017 | | | | | | | | | | | | |
| Iran | 9 | 1 | 69.11 (4.93) | NA | NA | NA | Control | NA | NA | NA | Post | BDNF |
| Iran | 11 | 1 | 68.81 (3.68) | 45 | Y | Y | AE + RE | 8 | 3 | 405 | Post | BDNF |
| Baharavar2025 | | | | | | | | | | | | |
| Iran | 12 | 1 | 64 (3) | NA | Y | NA | Control | NA | NA | NA | Post | BDNF |
| Iran | 12 | 1 | 65 (3) | 50 | Y | Y | AE + RE | 8 | 4 | 675 | 8 weeks | BDNF |
| Bo-RamKim2022 | | | | | | | | | | | | |
| South Korea | 9 | 1 | 67.78 (2.33) | NA | NA | NA | Control | NA | NA | NA | Post | BDNF |
| South Korea | 15 | 1 | 70.47 (5.57) | 60 | N | Y | AE | 12 | 4 | 810 | Post | BDNF |
| Bo-RamKim2023 | | | | | | | | | | | | |
| South Korea | 12 | 1 | 72.25 (5.07) | 60 | N | Y | RE | 12 | 4 | 720 | Post | BDNF |
| Byun2016 | | | | | | | | | | | | |
| South Korea | 11 | 1 | 70.46 (2.85) | NA | NA | NA | Control | NA | NA | NA | Post | BDNF |
| South Korea | 13 | 1 | 70.45 (4.18) | 50 | Y | Y | AE + RE | 12 | 5 | 1000 | Post | BDNF |
| Chou2022 | | | | | | | | | | | | |
| China | 28 | 1 | 69.8 (7.6) | NA | NA | NA | Control | NA | NA | NA | Post | BDNF |
| China | 30 | 1 | 69.3 (6.5) | 30 | N | Y | AE | 24 | 4 | 600 | Post | BDNF |
| ClareMaguire2023 | | | | | | | | | | | | |
| Switzerland | 10 | 1 | 60 (5) | 80 | N | Y | AE | 12 | 4 | 1080 | Post, 4 weeks, 12 weeks | BDNF |
| Switzerland | 7 | 1 | 56.6 (10.6) | 80 | N | Y | ME | 12 | 2 | 600 | Post, 4 weeks | BDNF |
| DeSá2024 | | | | | | | | | | | | |
| Brazil | 9 | 0.78 | 70 (6.7) | 60 | N | Y | AE + RE | 24 | 6 | 720 | Post | BDNF |
| Brazil | 9 | 0.78 | 70.3 (5.6) | 60 | N | Y | CE | 24 | 3 | 360 | Post | BDNF |
| Brazil | 9 | 0.78 | 73.66 (7.4) | 60 | N | Y | ME | 24 | 4 | 540 | Post | BDNF |
| Deus2021 | | | | | | | | | | | | |
| Brazil | 76 | 0.47 | 66.33 (3.88) | NA | NA | NA | Control | NA | NA | NA | Post | BDNF |
| Brazil | 81 | 0.43 | 67.27 (3.24) | 60 | N | Y | RE | 24 | 4 | 720 | Post | BDNF |
| Donyaei2023 | | | | | | | | | | | | |
| Iran | 17 | 1 | 62.1 (5.1) | NA | NA | NA | Control | NA | NA | NA | Post | BDNF |
| Iran | 17 | 1 | 61.3 (5.7) | 60 | N | Y | AE + RE | 12 | 6 | 1080 | 8 weeks, 12 weeks | BDNF |
| Enette2020 | | | | | | | | | | | | |
| France | 21 | 0.52 | 79.3 (2.3) | NA | NA | NA | Control | NA | NA | NA | Post | BDNF |
| France | 17 | 0.65 | 78.8 (1.8) | 30 | N | Y | AE | 9 | 6 | 330 | Post | BDNF |
| France | 14 | 0.79 | 74.8 (3.8) | 30 | N | Y | AE | 9 | 5 | 300 | Post | BDNF |
| Freeberg2024 | | | | | | | | | | | | |
| USA | 7 | 0.29 | 63 (6) | NA | NA | NA | Control | NA | NA | NA | Post | BDNF |
| USA | 9 | 0.44 | 65 (9) | 10 | Y | Y | RE | 6 | 5 | 300 | Post | BDNF |
| García-Salazar2024 | | | | | | | | | | | | |
| Brazil | 9 | 0.44 | 62 (20.5) | 40 | N | Y | AE | 2 | 6 | 480 | Post | BDNF |
| Brazil | 7 | 0.29 | 63 (14) | 40 | N | Y | CE | 2 | 3 | 240 | Post | BDNF |
| Ghodrati2023 | | | | | | | | | | | | |
| Iran | 9 | 1 | 55.8 (1.5) | NA | NA | NA | Control | NA | NA | NA | Post | BDNF |
| Iran | 12 | 1 | 58.8 (1.5) | 65 | N | Y | ME | 12 | 5 | 975 | Post | BDNF |
| HaritzArrieta2019 | | | | | | | | | | | | |
| Spain | 45 | 0.67 | 84.7 (6.1) | NA | NA | NA | Control | NA | NA | NA | Post | BDNF |
| Spain | 43 | 0.74 | 85.1 (7.6) | 60 | Y | Y | ME | 24 | 4 | 420 | Post | BDNF |
| Heissel2015 | | | | | | | | | | | | |
| Germany | 6 | 0.5 | 64.8 (9.1) | NA | NA | NA | Control | NA | NA | NA | Post | BDNF |
| Germany | 6 | 0.67 | 68.7 (3.1) | 60 | N | Y | ME | 4 | 4 | 480 | Post | BDNF |
| Hola2024 | | | | | | | | | | | | |
| Czech Republic | 23 | 0.82 | 70.1 (3.7) | NA | NA | NA | Control | NA | NA | NA | Post | BDNF |
| Czech Republic | 20 | 0.8 | 69.6 (3.5) | 90 | Y | Y | MBE | 12 | 4 | 810 | Post | BDNF |
| Czech Republic | 16 | 0.88 | 71.1 (4.3) | 90 | Y | Y | MBE | 12 | 5 | 900 | Post | BDNF |
| Hvid2017 | | | | | | | | | | | | |
| Denmark | 25 | 0.76 | 82.2 (4.5) | NA | NA | NA | Control | NA | NA | NA | Post | BDNF |
| Denmark | 22 | 0.55 | 82.7 (5.4) | 60 | Y | Y | RE | 12 | 5 | 600 | Post | BDNF |
| Kang2020 | | | | | | | | | | | | |
| South Korea | 10 | 1 | 73.37 (4.19) | NA | NA | NA | Control | NA | NA | NA | Post | BDNF |
| South Korea | 10 | 1 | 73.83 (3.95) | 60 | Y | Y | AQE | 16 | 4 | 810 | Post | BDNF |
| Khanthong2024 | | | | | | | | | | | | |
| Thailand | 29 | 0.85 | 60.93 (7.71) | NA | NA | NA | Control | NA | NA | NA | Post | BDNF |
| Thailand | 29 | 0.85 | 60.31 (5.69) | 60 | N | Y | MBE | 12 | 3 | 540 | 12 weeks | BDNF |
| Kim2018 | | | | | | | | | | | | |
| South Korea | 12 | 1 | 71.43 (4.45) | NA | NA | NA | Control | NA | NA | NA | Post | BDNF |
| South Korea | 14 | 1 | 71.77 (3.07) | 60 | Y | Y | AQE | 16 | 4 | 540 | Post | BDNF |
| Kirk2011 | | | | | | | | | | | | |
| USA | 60 | 0.6 | 65.5 (5.44) | NA | NA | NA | Control | NA | NA | NA | Post | BDNF |
| USA | 60 | 0.73 | 67.6 (5.81) | 40 | Y | Y | AE | 48 | 4 | 480 | Post | BDNF |
| Kohanpour2017 | | | | | | | | | | | | |
| Iran | 10 | 0 | 67.85 (3.89) | NA | NA | NA | Control | NA | NA | NA | Post | BDNF |
| Iran | 10 | 0 | 67.85 (3.89) | 50 | N | Y | AE | 13 | 6 | 900 | Post | BDNF |
| Kovacevic2020 | | | | | | | | | | | | |
| Canada | 22 | 0.65 | 71.5 (6.6) | NA | NA | NA | Control | NA | NA | NA | Post | BDNF |
| Canada | 19 | 0.5 | 72 (6.2) | 53 | Y | Y | AE | 12 | 4 | 716 | Post | BDNF |
| Canada | 19 | 0.67 | 72.4 (4.4) | 43 | Y | Y | HIIT | 12 | 8 | 1032 | Post | BDNF |
| Küster2017 | | | | | | | | | | | | |
| Germany | 18 | 0.5 | 69.9 (5.7) | NA | NA | NA | Control | NA | NA | NA | Post | BDNF |
| Germany | 17 | 0.68 | 73.2 (6) | 20 | N | Y | ME | 10 | 4 | 350 | Post | BDNF |
| LarissaJéssicadaSilva2024 | | | | | | | | | | | | |
| Brazil | 19 | 1 | 68.78 (4.56) | NA | NA | NA | Control | NA | NA | NA | Post | BDNF |
| Brazil | 19 | 1 | 67.71 (3.95) | 30 | N | Y | RE | 12 | 2 | 225 | Post | BDNF |
| Ledreux2019 | | | | | | | | | | | | |
| USA | 38 | 0.69 | 72.9 (5) | NA | NA | NA | Control | NA | NA | NA | Post | BDNF |
| USA | 36 | 0.69 | 72.9 (5) | 35 | Y | Y | ME | 5 | 4 | 700 | Post | BDNF |
| Maass2016 | | | | | | | | | | | | |
| Germany | 17 | 0.52 | 68.4 (4.3) | NA | NA | NA | Control | NA | NA | NA | Post | BDNF |
| Germany | 20 | 0.52 | 68.4 (4.3) | 40 | Y | Y | AE | 12 | 6 | 720 | Post | BDNF |
| MarenS2014 | | | | | | | | | | | | |
| USA | 14 | 0.6 | 67.4 (6.1) | NA | NA | NA | Control | NA | NA | NA | Post | BDNF |
| USA | 17 | 0.6 | 67.5 (5.6) | 60 | Y | Y | RE | 6 | 4 | 480 | Post | BDNF |
| Matura2017 | | | | | | | | | | | | |
| Germany | 24 | 0.52 | 77 (8.1) | NA | NA | NA | Control | NA | NA | NA | Post | BDNF |
| Germany | 29 | 0.42 | 73.3 (5.5) | 30 | Y | Y | AE | 12 | 5 | 450 | Post | BDNF |
| Nascimento2014 | | | | | | | | | | | | |
| Brazil | 15 | 0.67 | 68.1 (5.7) | NA | NA | NA | Control | NA | NA | NA | Post | BDNF |
| Brazil | 15 | 0.6 | 66.6 (7.9) | 60 | N | Y | ME | 16 | 5 | 900 | Post | BDNF |
| Osali2020 | | | | | | | | | | | | |
| Iran | 11 | 1 | 62.3 (1.23) | NA | NA | NA | Control | NA | NA | NA | Post | BDNF |
| Iran | 11 | 1 | 62.3 (1.23) | 50 | Y | Y | AE | 6 | 5 | 750 | Post | BDNF |
| Prestes2015 | | | | | | | | | | | | |
| Brazil | 10 | 1 | 66.9 (7.6) | NA | NA | NA | Control | NA | NA | NA | Post | BDNF |
| Brazil | 20 | 1 | 69.2 (6) | 50 | Y | Y | RE | 16 | 5 | 500 | Post | BDNF |
| Brazil | 20 | 1 | 65.5 (4.7) | 50 | Y | Y | RE | 16 | 5 | 500 | Post | BDNF |
| Rahmawati2024 | | | | | | | | | | | | |
| Indonesia | 15 | 1 | 71.27 (7.32) | 35 | Y | Y | AE | 8 | 2 | 210 | Post | BDNF |
| Indonesia | 15 | 1 | 69.53 (5.77) | 35 | Y | Y | CE | 8 | 5 | 525 | Post | BDNF |
| Rodziewicz2023 | | | | | | | | | | | | |
| Poland | 13 | 0.5 | 71.7 (5.5) | NA | NA | NA | Control | NA | NA | NA | Post | BDNF |
| Poland | 14 | 0.5 | 70.1 (4.5) | 50 | Y | Y | CE | 12 | 4 | 600 | Post | BDNF |
| Poland | 14 | 0.5 | 70.6 (5.3) | 50 | Y | Y | MBE | 12 | 4 | 600 | 12 weeks | BDNF |
| Ruiz2015 | | | | | | | | | | | | |
| Spain | 20 | 0.8 | 92.3 (2.3) | NA | NA | NA | Control | NA | NA | NA | Post | BDNF |
| Spain | 20 | 0.8 | 92.1 (2.3) | 40 | Y | Y | RE | 8 | 4 | 420 | Post | BDNF |
| Salisbury2023 | | | | | | | | | | | | |
| USA | 8 | 0.39 | 79.3 (5.5) | NA | NA | NA | Control | NA | NA | NA | Post | BDNF |
| USA | 18 | 0.25 | 76.8 (7.6) | 50 | N | Y | AE | 26 | 5 | 750 | 12 weeks, 24 weeks | BDNF |
| Singsanan2024 | | | | | | | | | | | | |
| Thailand | 20 | 1 | 61.6 (7) | NA | NA | NA | Control | NA | NA | NA | Post | BDNF |
| Thailand | 20 | 1 | 62.6 (4.5) | 60 | N | Y | MBE | 8 | 4 | 630 | Post | BDNF |
| Solianik2021 | | | | | | | | | | | | |
| Lithuania | 15 | 0.87 | 67 (5.9) | NA | NA | NA | Control | NA | NA | NA | Post | BDNF |
| Lithuania | 15 | 0.87 | 67.2 (5.9) | 60 | Y | Y | MBE | 10 | 3 | 360 | Post | BDNF |
| Tait2025 | | | | | | | | | | | | |
| Australia | 103 | 0.82 | 77.7 (7.2) | NA | NA | NA | Control | NA | NA | NA | Post | BDNF |
| Australia | 108 | 0.65 | 77.2 (6.6) | 52 | Y | Y | RE | 24 | 4 | 364 | 24 weeks | BDNF |
| Tarazona2016 | | | | | | | | | | | | |
| Spain | 49 | 0.51 | 80.3 (3.7) | NA | NA | NA | Control | NA | NA | NA | Post | BDNF |
| Spain | 51 | 0.57 | 79.7 (3.6) | 65 | Y | Y | ME | 24 | 5 | 1625 | Post | BDNF |
| Tsai2025 | | | | | | | | | | | | |
| China | 23 | 1 | 64.04 (5.65) | NA | NA | NA | Control | NA | NA | NA | Post | BDNF |
| China | 20 | 1 | 63.6 (3.84) | 30 | Y | Y | HIIT | 1 | 9 | 270 | Post | BDNF |
| China | 20 | 1 | 63.95 (4.41) | 30 | Y | Y | ME | 1 | 8 | 255 | Post | BDNF |
| Urzi2019 | | | | | | | | | | | | |
| Slovenia | 9 | 1 | 88.9 (5.3) | NA | NA | NA | Control | NA | NA | NA | Post | BDNF |
| Slovenia | 11 | 1 | 84.4 (7.7) | 60 | Y | Y | RE | 12 | 5 | 900 | Post | BDNF |
| Vaughan2014 | | | | | | | | | | | | |
| Australia | 24 | 1 | 68.8 (3.5) | NA | NA | NA | Control | NA | NA | NA | Post | BDNF |
| Australia | 25 | 1 | 69 (3.1) | 60 | Y | Y | ME | 16 | 5 | 600 | Post | BDNF |
| Vedovelli2017 | | | | | | | | | | | | |
| China | 9 | 1 | 77.3 (9.9) | NA | NA | NA | Control | NA | NA | NA | Post | BDNF |
| China | 20 | 1 | 83 (6.5) | 60 | Y | Y | AE + RE | 12 | 5 | 900 | Post | BDNF |
| XiLi2021 | | | | | | | | | | | | |
| China | 9 | 0.44 | 63.9 (3.95) | NA | NA | NA | Control | NA | NA | NA | Post | BDNF |
| China | 10 | 0.4 | 66.4 (4.5) | 45 | N | Y | AE | 12 | 6 | 810 | Post | BDNF |
| China | 10 | 0.3 | 64.9 (3.45) | 45 | N | Y | HIIT | 12 | 8 | 1080 | Post | BDNF |
| Zhang2023 | | | | | | | | | | | | |
| China | 10 | 0.93 | 69.75 (7.02) | NA | NA | NA | Control | NA | NA | NA | Post | BDNF |
| China | 13 | 0.93 | 66.22 (5.51) | 60 | N | Y | AE | 12 | 4 | 720 | Post | BDNF |
| China | 14 | 0.86 | 66.67 (6.04) | 60 | N | Y | CE | 12 | 5 | 900 | Post | BDNF |
| Čekanauskaitė2020 | | | | | | | | | | | | |
| Lithuania | 15 | 0.87 | 66.9 (6) | NA | NA | NA | Control | NA | NA | NA | Post | BDNF |
| Lithuania | 18 | 0.94 | 67.1 (6) | 90 | Y | Y | MBE | 10 | 2 | 450 | Post | BDNF |

#

# Appendix 3: Within-study risk of bias


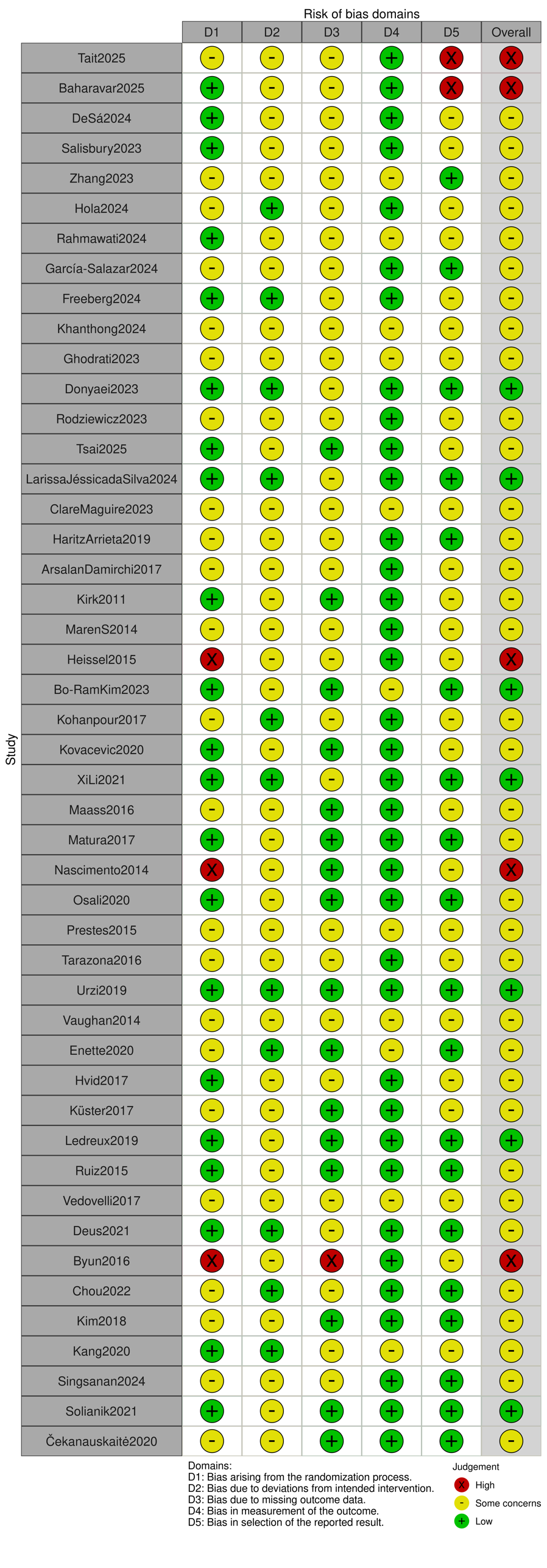


**Fig. S1**. Detailed risk-of-bias judgments for the included randomized controlled trials.


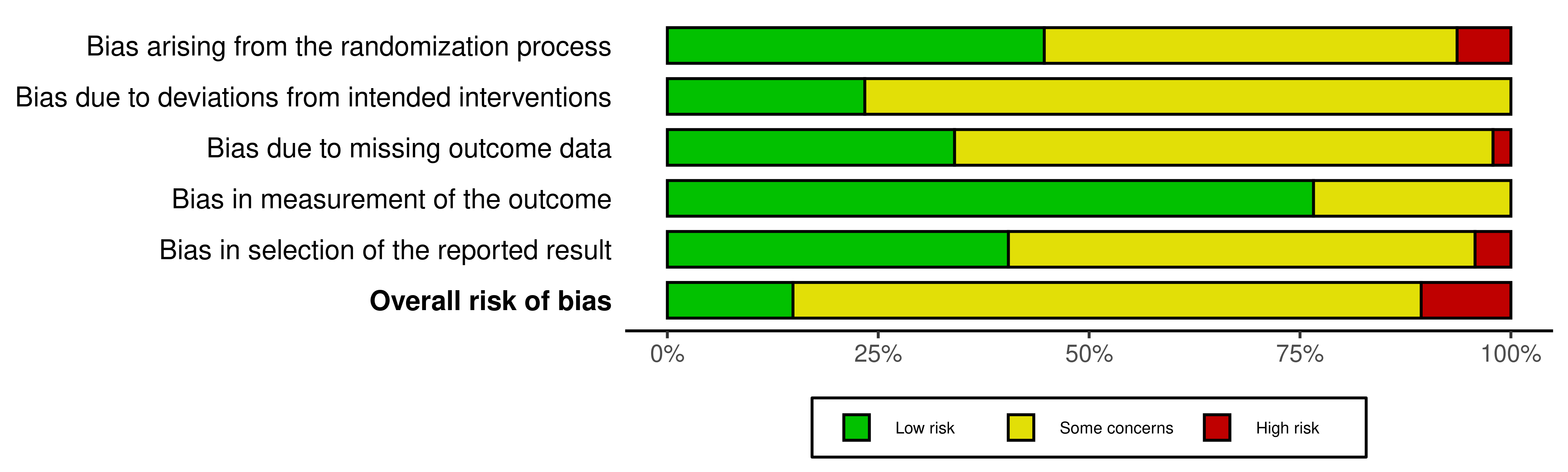


**Fig. S2**. Summary of risk-of-bias judgments across the included randomized controlled trials

# Appendix 4: Bayesian Network Meta-Analysis of Exercise and BDNF in Older Adults

**4.1 Posterior R² distributions of predictor models**


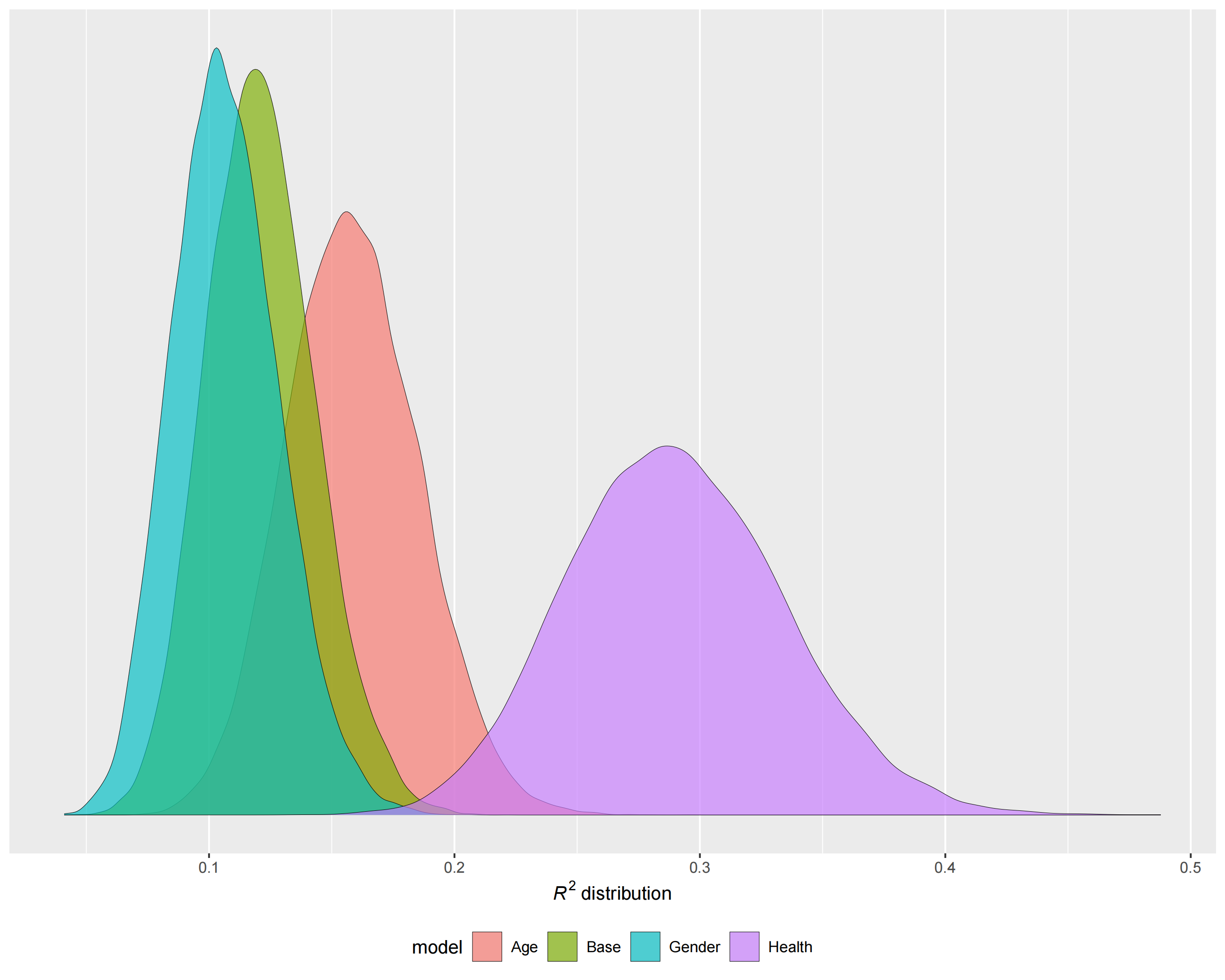


**Fig. S3**  Posterior Distributions of R² for Competing Models Including Age, Gender, Health, and Baseline Predictors

**4.2 Subgroup analysis of exercise effects on BDNF in healthy and unhealthy older adults**


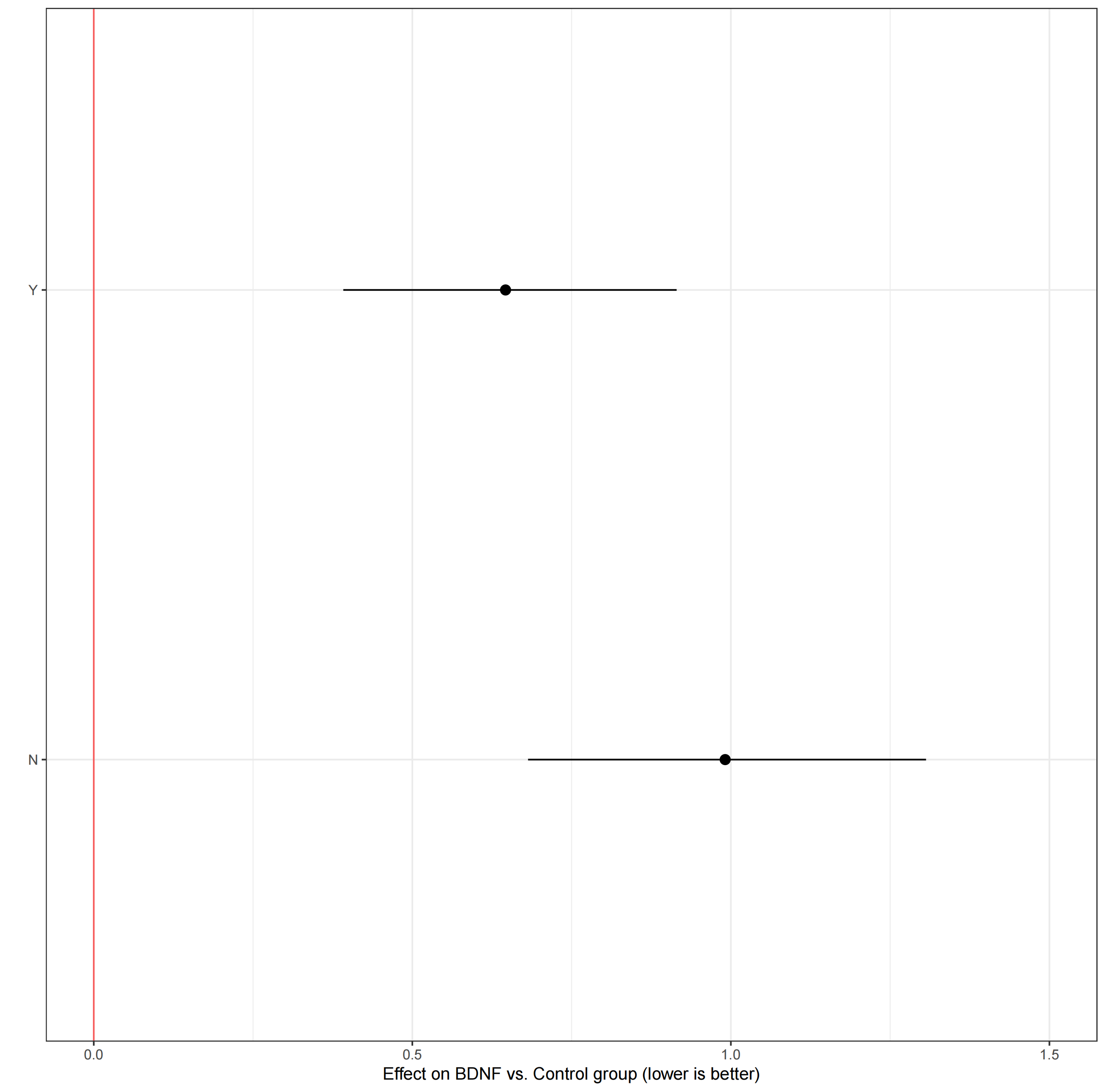
**Fig. S4 Results of Subgroup Analysis**

**4.3 Forest plot of study-level effects of exercise on BDNF**





**Fig. S5** Forest Plot of Study-Level Effect Sizes for the Impact of Exercise on BDNF Levels in Older Adults

**4.4 Sensitivity analyses and transitivity assessment**

### 4.4.1 Supplementary Table S1. Sensitivity analyses using different assumed pre–post correlation coefficients for change-score SD imputation.

| **Assumed pre-post correlation coefficient** | **Intervention** | **Hedges’ g** | **Lower 95% CrI** | **Upper 95% CrI** | **Interpretation** |
| --- | --- | --- | --- | --- | --- |
| 0.200 | AE | 0.274 | 0.085 | 0.463 | Positive effect |
| 0.200 | AE + RE | 0.626 | 0.277 | 0.973 | Positive effect |
| 0.200 | AQE | 1.014 | 0.340 | 1.669 | Positive effect |
| 0.200 | CE | 0.075 | -0.330 | 0.484 | Uncertain effect |
| 0.200 | HIIT | 0.283 | -0.141 | 0.709 | Uncertain effect |
| 0.200 | MBE | 0.656 | 0.341 | 0.975 | Positive effect |
| 0.200 | ME | 0.312 | 0.103 | 0.521 | Positive effect |
| 0.200 | RE | 0.428 | 0.236 | 0.619 | Positive effect |
| 0.500 | AE | 0.245 | 0.086 | 0.403 | Positive effect |
| 0.500 | AE + RE | 0.579 | 0.276 | 0.877 | Positive effect |
| 0.500 | AQE | 1.000 | 0.442 | 1.559 | Positive effect |
| 0.500 | CE | 0.109 | -0.231 | 0.449 | Uncertain effect |
| 0.500 | HIIT | 0.253 | -0.097 | 0.598 | Uncertain effect |
| 0.500 | MBE | 0.612 | 0.342 | 0.883 | Positive effect |
| 0.500 | ME | 0.305 | 0.135 | 0.475 | Positive effect |
| 0.500 | RE | 0.374 | 0.213 | 0.534 | Positive effect |
| 0.800 | AE | 0.204 | 0.095 | 0.312 | Positive effect |
| 0.800 | AE + RE | 0.523 | 0.307 | 0.741 | Positive effect |
| 0.800 | AQE | 0.954 | 0.526 | 1.378 | Positive effect |
| 0.800 | CE | 0.149 | -0.098 | 0.397 | Uncertain effect |
| 0.800 | HIIT | 0.204 | -0.038 | 0.443 | Uncertain effect |
| 0.800 | MBE | 0.503 | 0.296 | 0.710 | Positive effect |
| 0.800 | ME | 0.276 | 0.161 | 0.393 | Positive effect |
| 0.800 | RE | 0.229 | 0.118 | 0.340 | Positive effect |

**Note.** AE, aerobic exercise; AE + RE, combined aerobic and resistance exercise; AQE, aquatic exercise; CE, coordination exercise; HIIT, high-intensity interval training; MBE, mind-body exercise; ME, mixed exercise; RE, resistance exercise; CrI, credible interval. Positive effect was defined as a 95% CrI excluding zero in the positive direction.

### 4.4.2 Supplementary Table S2. Sensitivity analysis excluding studies rated as high risk of bias.

| **Analysis** | **Intervention** | **Hedges’ g** | **Lower 95% CrI** | **Upper 95% CrI** | **Interpretation** |
| --- | --- | --- | --- | --- | --- |
| Primary analysis | AE | 0.245 | 0.086 | 0.403 | Positive effect |
| Primary analysis | AE + RE | 0.579 | 0.276 | 0.877 | Positive effect |
| Primary analysis | AQE | 1.000 | 0.442 | 1.559 | Positive effect |
| Primary analysis | CE | 0.109 | -0.231 | 0.449 | Uncertain effect |
| Primary analysis | HIIT | 0.253 | -0.097 | 0.598 | Uncertain effect |
| Primary analysis | MBE | 0.612 | 0.342 | 0.883 | Positive effect |
| Primary analysis | ME | 0.305 | 0.135 | 0.475 | Positive effect |
| Primary analysis | RE | 0.374 | 0.213 | 0.534 | Positive effect |
| High-risk excluded | AE | 0.254 | 0.095 | 0.414 | Positive effect |
| High-risk excluded | AE + RE | 0.474 | 0.113 | 0.835 | Positive effect |
| High-risk excluded | AQE | 1.012 | 0.443 | 1.581 | Positive effect |
| High-risk excluded | CE | 0.124 | -0.222 | 0.469 | Uncertain effect |
| High-risk excluded | HIIT | 0.260 | -0.091 | 0.616 | Uncertain effect |
| High-risk excluded | MBE | 0.629 | 0.357 | 0.902 | Positive effect |
| High-risk excluded | ME | 0.311 | 0.130 | 0.494 | Positive effect |
| High-risk excluded | RE | 0.681 | 0.475 | 0.884 | Positive effect |

**Note.** AE, aerobic exercise; AE + RE, combined aerobic and resistance exercise; AQE, aquatic exercise; CE, coordination exercise; HIIT, high-intensity interval training; MBE, mind-body exercise; ME, mixed exercise; RE, resistance exercise; CrI, credible interval. High-risk excluded indicates the sensitivity analysis excluding Tait2025, Baharavar2025, Heissel2015, Nascimento2014, and Byun2016. Positive effect was defined as a 95% CrI excluding zero in the positive direction.

### 4.4.3 Supplementary Table S3. Comparator/control classification across included studies

**Summary of final comparator categories**

| **Final comparator category** | **No. of studies** | **Active comparator?** | **Handling in active-comparator-exclusion sensitivity analysis** |
| --- | --- | --- | --- |
| Inactive control | 25 | No | Included |
| Usual care / standard treatment | 4 | No | Included |
| Active non-exercise control | 8 | Yes | Excluded |
| Active physical control | 6 | Yes | Excluded |
| No non-exercise control arm | 4 | NA | Separate handling; original exercise–exercise comparison structure retained |

**Detailed study-level classification**

| **Study / analysis ID / linked arms** | **Comparator evidence from full text** | **Final category and sensitivity handling** | **Classification rationale and notes** |
| --- | --- | --- | --- |
| **No.** 1  **Study ID:** Tait2025  **Analysis-level ID(s):** Tait2025  **Linked intervention arms:** RE | **Comparator arm label:** Control / usual care control  **Full-text comparator description:** Usual care control group. Participants maintained normal physical activity habits, received usual care from their medical practitioner and community services, and received falls-prevention / active-health booklets plus pamphlet-based encouragement to achieve the recommended 150 min/week physical activity.  **Original wording / direct quote:** “maintained their normal physical activity habits, and received usual care from their medical practitioner and community services” / “given a falls prevention booklet … and encouraged to achieve the recommended 150 min of weekly physical activity”  **Source page/section:** Methods; Study design; Control group | **Final comparator category:** Usual care / standard treatment  **Active comparator?** No  **Included in active-comparator-exclusion sensitivity analysis?** Yes | **Rationale for classification:** Comparator received usual care and maintained normal physical activity habits without a supervised, structured exercise comparator program.  **Notes / uncertainty:** Not a pure inactive control because the control arm received usual care plus booklets/pamphlet-based physical-activity encouragement. However, no structured exercise sessions, stretching/flexibility training, supervised physical activity, attention-matched education sessions, or active physical comparator was reported. |
| **No.** 2  **Study ID:** Baharavar2025  **Analysis-level ID(s):** Baharavar2025  **Linked intervention arms:** AE + RE | **Comparator arm label:** Control / C group  **Full-text comparator description:** Control group did not receive any intervention and followed their usual daily activities during the 8-week training period. Participants were also asked to maintain their usual daily activities and diet during the study.  **Original wording / direct quote:** “Participants in the C group did not received any intervention” / “C group did not participate in any intervention and they followed their usual daily activities during the training protocol.”  **Source page/section:** Abstract; Method; training protocol description | **Final comparator category:** Inactive control  **Active comparator?** No  **Included in active-comparator-exclusion sensitivity analysis?** Yes | **Rationale for classification:** Comparator received no structured intervention and followed usual daily activities.  **Notes / uncertainty:** No routine care, health education, social activity, attention control, stretching/flexibility comparator, or sham physical activity was reported for the control group. |
| **No.** 3  **Study ID:** DeSá2024  **Analysis-level ID(s):** DeSá2024  **Linked intervention arms:** AE + RE, CE, ME | **Comparator arm label:** No non-exercise control arm  **Full-text comparator description:** No non-exercise control arm was included. Participants were randomized into three active intervention groups: Physical Exercises, Motor Tasks, and Physical Exercises associated with Motor Tasks.  **Original wording / direct quote:** “three intervention groups: Physical Exercises (PE); Motor Tasks (MT); and Physical exercise combined with the motor task (PE+MT)” / “All groups performed the activities for 60 min per session, twice a week … for 6 months.”  **Source page/section:** Method; Randomization; Interventions | **Final comparator category:** No non-exercise control arm  **Active comparator?** NA  **Included in active-comparator-exclusion sensitivity analysis?** Separate handling | **Rationale for classification:** Study included only active exercise/motor-task intervention arms and no inactive, usual-care, or non-exercise control arm.  **Notes / uncertainty:** The MT arm involved an active motor-task protocol; therefore, it was handled as an exercise/motor-task comparison rather than an inactive or usual-care comparator. |
| **No.** 4  **Study ID:** Salisbury2023  **Analysis-level ID(s):** Salisbury2023  **Linked intervention arms:** AE | **Comparator arm label:** Control / stretching control  **Full-text comparator description:** Stretching control group. Participants performed supervised range-of-motion and flexibility training prescribed as light intensity, with session frequency and duration matched to the aerobic exercise group.  **Original wording / direct quote:** “Range of motion and flexibility training was prescribed as light (i.e., HRR ≤ 20%, RPE ≤ 8) with session frequency and duration matched to AEx group.”  **Source page/section:** Methods; Experimental Protocol; Stretching | **Final comparator category:** Active physical control  **Active comparator?** Yes  **Included in active-comparator-exclusion sensitivity analysis?** No | **Rationale for classification:** Comparator involved structured range-of-motion/flexibility training with physical activity components, even though it was prescribed at light intensity.  **Notes / uncertainty:** The comparator included structured stretching/flexibility content with matched frequency and duration; therefore, it was not treated as an inactive/no-intervention control. |
| **No.** 5  **Study ID:** Zhang2023  **Analysis-level ID(s):** Zhang2023  **Linked intervention arms:** AE, CE | **Comparator arm label:** Control / CG  **Full-text comparator description:** Control group attended education classes. In addition, participants in both intervention groups and the control group participated in monthly health science knowledge dissemination activities covering CI-related diseases, memory care, seasonal disease prevention, and prevention of common chronic diseases in older adults.  **Original wording / direct quote:** “the control group attended 3 education classes” / “all participants in the intervention and CG participated in monthly health science knowledge dissemination activities”  **Source page/section:** Abstract; Materials and Methods; Intervention | **Final comparator category:** Active non-exercise control  **Active comparator?** Yes  **Included in active-comparator-exclusion sensitivity analysis?** No | **Rationale for classification:** Comparator involved education/health-science activities but no structured physical activity or exercise training.  **Notes / uncertainty:** The comparator included education/health-science contact; therefore, it was handled as an active non-exercise comparator rather than an inactive control. |
| **No.** 6  **Study ID:** Hola2024  **Analysis-level ID(s):** Hola2024  **Linked intervention arms:** MBE | **Comparator arm label:** Control / CG  **Full-text comparator description:** Waitlist/usual-lifestyle control group. Control participants were placed on a waiting list and asked to maintain their usual lifestyle; no specific recommendations for physical activity were required.  **Original wording / direct quote:** “participants were either placed on a waiting list” / “participants were asked to maintain their usual lifestyle … no specific recommendations for physical activity were required”  **Source page/section:** Methods; Trial Design; Control Group | **Final comparator category:** Inactive control  **Active comparator?** No  **Included in active-comparator-exclusion sensitivity analysis?** Yes | **Rationale for classification:** Comparator was a waitlist/usual-lifestyle control without structured intervention, exercise sessions, education sessions, or physical activity recommendations.  **Notes / uncertainty:** The post-trial option to join dance or martial arts, or receive financial reward, was a participation benefit and not an active intervention during the study period. |
| **No.** 7  **Study ID:** Rahmawati2024  **Analysis-level ID(s):** Rahmawati2024  **Linked intervention arms:** AE; CE | **Comparator arm label:** AE / light-intensity aerobic exercise comparator  **Full-text comparator description:** No non-exercise control arm was included in the analysis-level comparison. The comparison was between two exercise-coded arms: light-intensity aerobic exercise and boxing exergame added to light-intensity aerobic exercise. The AE arm involved light-intensity aerobic activities and physical stretching, while the CE arm added boxing exergame training.  **Original wording / direct quote:** “Participants in both groups were required to participate in light intensity aerobic exercise five times a week for ±15 minutes.” / “Both groups are required to take part in light intensity aerobic exercise … in the form of a combination of light intensity aerobic activities and physical stretching.”  **Source page/section:** Abstract; Materials and Methods; Exercise intervention | **Final comparator category:** No non-exercise control arm  **Active comparator?** NA  **Included in active-comparator-exclusion sensitivity analysis?** Separate handling | **Rationale for classification:** Study compared active exercise/intervention arms and did not include an inactive, usual-care, or non-exercise control arm.  **Notes / uncertainty:** Because both arms contained exercise/physical-activity components and no separate non-exercise control arm was available for the NMA, this study was handled as an exercise–exercise comparison rather than as part of the comparator/control node. |
| **No.** 8  **Study ID:** García-Salazar2024  **Analysis-level ID(s):** García-Salazar2024  **Linked intervention arms:** AE; CE | **Comparator arm label:** CE / mCIMT + stretching comparator  **Full-text comparator description:** No non-exercise control arm was included in the analysis-level comparison. The comparison was between two active intervention arms: mCIMT with stretching and aerobic exercise plus mCIMT. The CE comparator arm included stretching exercises combined with modified constraint-induced movement therapy.  **Original wording / direct quote:** “control group that performed stretching exercises combined with mCIMT” / “Stretching was performed bilaterally in the upper and lower limbs…”  **Source page/section:** Methods; Intervention | **Final comparator category:** No non-exercise control arm  **Active comparator?** NA  **Included in active-comparator-exclusion sensitivity analysis?** Separate handling | **Rationale for classification:** Study compared active rehabilitation/exercise-based intervention arms and did not include an inactive, usual-care, or non-exercise control arm.  **Notes / uncertainty:** Although the article used the term control group, both arms represented active rehabilitation/exercise-based interventions in the analysis; therefore, it was handled as an exercise–exercise comparison rather than as an active control merged into the comparator node. |
| **No.** 9  **Study ID:** Freeberg2024  **Analysis-level ID(s):** Freeberg2024  **Linked intervention arms:** RE | **Comparator arm label:** Control / low-resistance Sham training  **Full-text comparator description:** Comparator group received low-resistance sham inspiratory muscle strength training at 15% maximal inspiratory pressure, using the same PowerBreathe device structure. Both groups performed 30 resisted inspirations per day, 6 days per week, for 6 weeks.  **Original wording / direct quote:** “low-resistance Sham training (15% PIMAX; 6x/week)” / “Both groups performed 30 resisted inspirations/day, 6 days/week … for 6 weeks.”  **Source page/section:** Abstract; Methods | **Final comparator category:** Active physical control  **Active comparator?** Yes  **Included in active-comparator-exclusion sensitivity analysis?** No | **Rationale for classification:** Comparator involved structured low-resistance respiratory muscle training with repeated resisted breathing efforts, despite being labelled sham.  **Notes / uncertainty:** Not inactive control or usual care. Although low-resistance and intended as sham, it still involved repeated resisted inspirations and matched training frequency. |
| **No.** 10  **Study ID:** Khanthong2024  **Analysis-level ID(s):** Khanthong2024  **Linked intervention arms:** MBE | **Comparator arm label:** Control / no intervention  **Full-text comparator description:** Control group received no structured intervention during the 12-week period. Both RD and control groups received baseline information regarding MCI/AD symptoms and lifestyle adjustment on the first day.  **Original wording / direct quote:** “The control group received no intervention.” / “both groups were given information regarding MCI symptoms by the physician on the first day.”  **Source page/section:** Abstract; Materials and Methods; Intervention; Figure 1 | **Final comparator category:** Inactive control  **Active comparator?** No  **Included in active-comparator-exclusion sensitivity analysis?** Yes | **Rationale for classification:** Comparator received no structured intervention or exercise program during follow-up.  **Notes / uncertainty:** Not a pure no-contact control because both groups received one-time baseline information, but this was not a structured health education or attention-control program. Therefore classify as Inactive control. |
| **No.** 11  **Study ID:** Ghodrati2023  **Analysis-level ID(s):** Ghodrati2023  **Linked intervention arms:** ME | **Comparator arm label:** Control / no-exercise control  **Full-text comparator description:** Control group received no exercise training intervention during the 12-week intervention period and was instructed not to begin a structured exercise program.  **Original wording / direct quote:** “the control group received no training intervention” / “did not receive an exercise intervention and were instructed not to begin a structured exercise program”  **Source page/section:** Abstract; Methods; Design | **Final comparator category:** Inactive control  **Active comparator?** No  **Included in active-comparator-exclusion sensitivity analysis?** Yes | **Rationale for classification:** Comparator received no structured intervention and was instructed not to start structured exercise.  **Notes / uncertainty:** Comparator classification concerned the control condition only; the intervention arm remained coded as ME in the NMA. |
| **No.** 12  **Study ID:** Donyaei2023  **Analysis-level ID(s):** Donyaei2023  **Linked intervention arms:** AE + RE | **Comparator arm label:** Control / usual lifestyle without exercise training  **Full-text comparator description:** Control group was instructed to follow usual diet and not make significant lifestyle changes during the 20-week study period. The experimental group performed 12 weeks of combined training followed by detraining.  **Original wording / direct quote:** “Each group was instructed to follow their usual diet.” / “The control group was also asked not to make any significant changes to their lifestyle during the 20 weeks of the study.”  **Source page/section:** Methods; Randomization; Intervention | **Final comparator category:** Inactive control  **Active comparator?** No  **Included in active-comparator-exclusion sensitivity analysis?** Yes | **Rationale for classification:** Comparator received no structured exercise intervention and was asked to maintain usual lifestyle.  **Notes / uncertainty:** No health education, attention-control activity, stretching, sham exercise, or structured physical comparator was reported for the control group. |
| **No.** 13  **Study ID:** Rodziewicz2023  **Analysis-level ID(s):** Rodziewicz2023  **Linked intervention arms:** CE, MBE | **Comparator arm label:** Control / no training intervention  **Full-text comparator description:** Control group did not participate in any training intervention and was instructed not to change daily habits during the intervention phase.  **Original wording / direct quote:** “The control group did not participate in any training intervention.” / “Participants were instructed not to change their daily habits during the intervention phase.”  **Source page/section:** Materials and methods; Study design; Control group | **Final comparator category:** Inactive control  **Active comparator?** No  **Included in active-comparator-exclusion sensitivity analysis?** Yes | **Rationale for classification:** Comparator received no structured training and maintained daily habits.  **Notes / uncertainty:** The same inactive control arm was linked to two active intervention arms in the analysis-level dataset. |
| **No.** 14  **Study ID:** Tsai2025  **Analysis-level ID(s):** Tsai2025  **Linked intervention arms:** HIIT, ME | **Comparator arm label:** Control / resting session  **Full-text comparator description:** Control group underwent a non-exercise intervention session consisting of resting while sitting and reading magazines for 32 minutes.  **Original wording / direct quote:** “non-exercise-intervention group (Control)” / “spent 32 min resting, by sitting and reading magazines”  **Source page/section:** Methods; Participants; Control group | **Final comparator category:** Inactive control  **Active comparator?** No  **Included in active-comparator-exclusion sensitivity analysis?** Yes | **Rationale for classification:** Comparator was a non-exercise resting condition without structured training.  **Notes / uncertainty:** Reading magazines occurred during the resting comparator session and was not classified as structured cognitive training, health education, or an attention-control intervention. |
| **No.** 15  **Study ID:** de Souza HCM 2024  **Analysis-level ID(s):** LarissaJéssicadaSilva2024  **Linked intervention arms:** RE | **Comparator arm label:** Control / sham group  **Full-text comparator description:** Comparator was the sham group. It received inspiratory muscle training with a low fixed load and was positioned on the vibratory platform without therapeutic effect.  **Original wording / direct quote:** “Sham groups (G3)” / “G3 received IMT with a low fixed load and were positioned at the vibratory platform without therapeutic effect.”  **Source page/section:** Abstract; Methods; Intervention protocol | **Final comparator category:** Active physical control  **Active comparator?** Yes  **Included in active-comparator-exclusion sensitivity analysis?** No | **Rationale for classification:** Comparator involved sham physical procedures, including low-load inspiratory muscle training and positioning on a vibratory platform.  **Notes / uncertainty:** The analysis-level study ID was retained as LarissaJéssicadaSilva2024 for merge consistency. Participants in the comparator arm still performed sham/low-load physical procedures; therefore, the arm was not classified as inactive control. |
| **No.** 16  **Study ID:** ClareMaguire2023  **Analysis-level ID(s):** ClareMaguire2023  **Linked intervention arms:** AE, ME | **Comparator arm label:** No non-exercise control arm / LI-CT + RT active comparator  **Full-text comparator description:** No non-exercise control arm was included. Participants were randomized to moderate-intensity cycle-ergometer training plus upper-limb robotic/sensor-based training, or low-intensity gait and balance circuit training plus upper-limb robotic/sensor-based training.  **Original wording / direct quote:** “randomized into: (1) MI-ET&RT or (2) LI-CT&RT” / “Both groups completed 40 mins MI-ET or LI-CT followed by 40 mins RT.”  **Source page/section:** Abstract; Methods; Study arms; Interventions | **Final comparator category:** No non-exercise control arm  **Active comparator?** NA  **Included in active-comparator-exclusion sensitivity analysis?** Separate handling | **Rationale for classification:** Study compared two active rehabilitation/exercise-based intervention arms and did not include inactive, usual-care, or non-exercise control.  **Notes / uncertainty:** LI-CT + RT was not treated as a shared non-exercise comparator/control node; active arms were retained as AE and ME in the network. |
| **No.** 17  **Study ID:** HaritzArrieta2019  **Analysis-level ID(s):** HaritzArrieta2019  **Linked intervention arms:** ME | **Comparator arm label:** Control / routine nursing-home activities  **Full-text comparator description:** Control group participated in routine activities offered by the nursing homes, including memory workshops, reading, singing, and similar activities.  **Original wording / direct quote:** “participated in routine activities that the nursing homes offered to residents: memory workshops, reading, singing, and other similar activities.”  **Source page/section:** Methods; Control group | **Final comparator category:** Active non-exercise control  **Active comparator?** Yes  **Included in active-comparator-exclusion sensitivity analysis?** No | **Rationale for classification:** Comparator involved routine cognitive/social activities without structured physical exercise.  **Notes / uncertainty:** This is not a pure inactive/no-intervention control because memory workshops, reading, and singing were provided as routine activities. Conservative classification as Active non-exercise control is appropriate. |
| **No.** 18  **Study ID:** ArsalanDamirchi2017  **Analysis-level ID(s):** ArsalanDamirchi2017  **Linked intervention arms:** AE + RE | **Comparator arm label:** Control / waitlist control  **Full-text comparator description:** Control group was assigned to a waitlist. The trial also included physical training, mental training, and combined training groups, but the analysis retained the physical-training comparison against control.  **Original wording / direct quote:** “Assigned to wait list (Control Group)” / “physical training (PH), mental training (ME), PH + ME, and control group (CO)”  **Source page/section:** Methods; Participants; Figure 1 | **Final comparator category:** Inactive control  **Active comparator?** No  **Included in active-comparator-exclusion sensitivity analysis?** Yes | **Rationale for classification:** Comparator was a waitlist control without structured intervention.  **Notes / uncertainty:** The original article included four arms; the analysis-level coding retained the relevant physical-training comparison as AE + RE. |
| **No.** 19  **Study ID:** Kirk2011  **Analysis-level ID(s):** Kirk2011  **Linked intervention arms:** AE | **Comparator arm label:** Control / stretching and toning control  **Full-text comparator description:** Stretching and toning control group attended supervised classes that included warm-up and cool-down stretching, muscle-toning exercises using dumbbells or resistance bands, balance exercises, a yoga sequence, exercise logs, and feedback.  **Original wording / direct quote:** “stretching and toning exercises that served as a control” / “muscle-toning exercises using dumbbells or resistance bands, two exercises designed to improve balance, one yoga sequence”  **Source page/section:** Methods; Training Protocol; Stretching and toning control condition | **Final comparator category:** Active physical control  **Active comparator?** Yes  **Included in active-comparator-exclusion sensitivity analysis?** No | **Rationale for classification:** Comparator involved structured stretching, toning, balance, and yoga-like physical activity components.  **Notes / uncertainty:** Although this arm served as the trial control, it was classified as an active physical comparator because it included structured stretching/toning sessions. |
| **No.** 20  **Study ID:** MarenS2014  **Analysis-level ID(s):** MarenS2014  **Linked intervention arms:** RE | **Comparator arm label:** Control / no intervention  **Full-text comparator description:** Control group received no intervention during the 6-week period; outcomes were measured before and after resistance training or no-intervention control.  **Original wording / direct quote:** “after 6 weeks of resistance training or no intervention (control)” / “randomized into the training or control group”  **Source page/section:** Methods; Experimental Approach; Subjects | **Final comparator category:** Inactive control  **Active comparator?** No  **Included in active-comparator-exclusion sensitivity analysis?** Yes | **Rationale for classification:** Comparator received no structured intervention.  **Notes / uncertainty:** This is a straightforward no-intervention control. R studyID is retained as MarenS2014. |
| **No.** 21  **Study ID:** Heissel2015  **Analysis-level ID(s):** Heissel2015  **Linked intervention arms:** ME | **Comparator arm label:** ACG / active control group  **Full-text comparator description:** Active control group received relaxation-based sessions twice weekly for four weeks, matched in frequency and duration to the physical exercise group. Sessions included warm-up trust exercises, relaxation tasks, progressive muscle relaxation, and end-session stretching.  **Original wording / direct quote:** “active control group (ACG; n = 6; relaxation exercises)” / “patients practiced the method of progressive muscle relaxation” / “Each session ended with stretching”  **Source page/section:** Abstract; Methods; Interventions | **Final comparator category:** Active non-exercise control  **Active comparator?** Yes  **Included in active-comparator-exclusion sensitivity analysis?** No | **Rationale for classification:** Comparator was primarily a structured relaxation/attention-control intervention rather than no intervention or usual lifestyle.  **Notes / uncertainty:** The comparator was classified as active non-exercise because the main comparator content was relaxation/PMR and the authors stated that activities were kept below the threshold for physical training. Stretching appeared adjunctive. This study was excluded from the active-comparator-exclusion sensitivity analysis. |
| **No.** 22  **Study ID:** Bo-RamKim2022; Bo-RamKim2023  **Analysis-level ID(s):** Bo-RamKim2022; Bo-RamKim2023  **Linked intervention arms:** AE; RE | **Comparator arm label:** Control / CG  **Full-text comparator description:** Control group was one of three randomized arms together with aerobic exercise and resistance exercise groups. The paper describes the two physical activity interventions, while no structured exercise, health education, placebo/sham, stretching, or other active comparator content was reported for the control group.  **Original wording / direct quote:** “The participants were randomly assigned into the aerobic exercise group … resistance exercise group … and control group.” / “The study subjects did not participate in a regular structured aerobic and resistance exercise program for at least four months prior to this study.”  **Source page/section:** Methods; Participation | **Final comparator category:** Inactive control  **Active comparator?** No  **Included in active-comparator-exclusion sensitivity analysis?** Yes | **Rationale for classification:** Comparator appears to be a non-exercise control arm, with no additional structured comparator intervention reported.  **Notes / uncertainty:** Control protocol details remain limited because the paper does not provide a separate detailed CG protocol. However, the article only describes AE and RE as the intervention arms and does not report any active comparator content for CG. Therefore, retaining Inactive control is appropriate. |
| **No.** 23  **Study ID:** Kohanpour2017  **Analysis-level ID(s):** Kohanpour2017  **Linked intervention arms:** AE | **Comparator arm label:** Placebo group  **Full-text comparator description:** Placebo group received placebo essence prepared by a pharmacist at a concentration without therapeutic effect. The trial had four groups: aerobic exercise, lavender extract, aerobic exercise plus lavender, and placebo.  **Original wording / direct quote:** “randomly divided into 4 groups: aerobic exercise, lavender extract, aerobic-lavender, and placebo” / “The placebo was made by pharmacist … such that it did not possess any therapeutic effect.”  **Source page/section:** Methods | **Final comparator category:** Active non-exercise control  **Active comparator?** Yes  **Included in active-comparator-exclusion sensitivity analysis?** No | **Rationale for classification:** Comparator involved a placebo/sham non-exercise intervention without structured physical activity.  **Notes / uncertainty:** The placebo intervention meant that this arm was not classified as inactive control; it was excluded from the active-comparator-exclusion sensitivity analysis. |
| **No.** 24  **Study ID:** Kovacevic2020  **Analysis-level ID(s):** Kovacevic2020  **Linked intervention arms:** AE, HIIT | **Comparator arm label:** CON / stretching control  **Full-text comparator description:** Stretching control group was used as the comparator against high-intensity interval training and moderate continuous training. Participants were assigned to HIIT, MCT, or stretching control.  **Original wording / direct quote:** “high-intensity interval training (HIIT); moderate continuous training (MCT); or stretching control (CON)”  **Source page/section:** Abstract; Materials and Methods | **Final comparator category:** Active physical control  **Active comparator?** Yes  **Included in active-comparator-exclusion sensitivity analysis?** No | **Rationale for classification:** Comparator involved stretching, which is a structured physical activity component.  **Notes / uncertainty:** The stretching condition was classified as an active physical comparator rather than an inactive control. |
| **No.** 25  **Study ID:** XiLi2021  **Analysis-level ID(s):** XiLi2021  **Linked intervention arms:** AE, HIIT | **Comparator arm label:** CON / physically inactive control group  **Full-text comparator description:** Control group was physically inactive and lived normally without participating in HIIT or VICT training protocols during the 12-week period.  **Original wording / direct quote:** “randomly divided into a control group (CON), an HIIT group … and a VICT group” / “The subjects in the CON group lived normally…”  **Source page/section:** Abstract; Methods; Intervention | **Final comparator category:** Inactive control  **Active comparator?** No  **Included in active-comparator-exclusion sensitivity analysis?** Yes | **Rationale for classification:** Comparator received no structured exercise training and maintained normal living.  **Notes / uncertainty:** This is an inactive/non-training comparator. No health education, placebo, stretching, sham exercise, or other active comparator was reported for CON. |
| **No.** 26  **Study ID:** Maass2016  **Analysis-level ID(s):** Maass2016  **Linked intervention arms:** AE | **Comparator arm label:** Control / progressive muscle relaxation-stretching  **Full-text comparator description:** Control group received 45 min of supervised progressive muscle relaxation/stretching training twice weekly. It was selected to match social interaction, schedule, and motivation while not affecting cardiovascular fitness.  **Original wording / direct quote:** “progressive muscle relaxation/stretching (control group)” / “received 45 min of supervised progressive muscle relaxation/stretching training … twice a week”  **Source page/section:** Methods; Progressive muscle relaxation/stretching control group | **Final comparator category:** Active non-exercise control  **Active comparator?** Yes  **Included in active-comparator-exclusion sensitivity analysis?** No | **Rationale for classification:** Comparator involved a structured relaxation/attention-matched control intervention rather than no intervention or usual lifestyle.  **Notes / uncertainty:** Although stretching is mentioned, the comparator was primarily progressive muscle relaxation and was chosen not to affect cardiovascular fitness. Therefore classify as active non-exercise rather than inactive control. |
| **No.** 27  **Study ID:** Matura2017  **Analysis-level ID(s):** Matura2017  **Linked intervention arms:** AE | **Comparator arm label:** Waiting control group  **Full-text comparator description:** Waiting control group waited 12 weeks before starting exercise and was asked not to change habitual physical activity during the 3-month period.  **Original wording / direct quote:** “waiting control group” / “waited 12 weeks before starting the exercise intervention” / “asked not to change their habitual physical activity”  **Source page/section:** Materials and Methods; Design; Setting | **Final comparator category:** Inactive control  **Active comparator?** No  **Included in active-comparator-exclusion sensitivity analysis?** Yes | **Rationale for classification:** Comparator was a waiting-list/no-exercise control with maintenance of habitual physical activity.  **Notes / uncertainty:** Later delayed exercise for the waiting group occurred after the comparison period and should not be considered active comparator during the main intervention period. |
| **No.** 28  **Study ID:** Nascimento2014  **Analysis-level ID(s):** Nascimento2014  **Linked intervention arms:** ME | **Comparator arm label:** Control / no regular physical exercise program  **Full-text comparator description:** Control groups did not attend any regular physical exercise program for at least one year before the study and throughout the research period.  **Original wording / direct quote:** “control groups did not attend to any kind of regular physical exercises program … throughout the longstanding research”  **Source page/section:** Methods; Participants / group allocation | **Final comparator category:** Inactive control  **Active comparator?** No  **Included in active-comparator-exclusion sensitivity analysis?** Yes | **Rationale for classification:** Comparator received no regular structured physical exercise program during the research period.  **Notes / uncertainty:** The study included control groups for both cognitively normal and MCI participants; the linked analysis-level intervention was ME and the shared control was classified as inactive. |
| **No.** 29  **Study ID:** Osali2020  **Analysis-level ID(s):** Osali2020  **Linked intervention arms:** AE | **Comparator arm label:** Placebo / MetS control  **Full-text comparator description:** Control group was the placebo condition. Participants were assigned to placebo control, aerobic exercise, nano-curcumin supplementation, or combined aerobic exercise plus nano-curcumin. Placebo participants received maltodextrin capsules identical in appearance, size, and taste.  **Original wording / direct quote:** “placebo (control)” / “randomly divided into 4 groups … MetS control (MC)” / “placebo (80 mg per day maltodextrin)”  **Source page/section:** Materials and methods; Main trials | **Final comparator category:** Active non-exercise control  **Active comparator?** Yes  **Included in active-comparator-exclusion sensitivity analysis?** No | **Rationale for classification:** Comparator involved a placebo non-exercise intervention without structured physical activity.  **Notes / uncertainty:** The placebo capsules constituted active non-exercise comparator content; this study was excluded from the active-comparator-exclusion sensitivity analysis. |
| **No.** 30  **Study ID:** Prestes2015  **Analysis-level ID(s):** Prestes2015  **Linked intervention arms:** RE | **Comparator arm label:** Control group  **Full-text comparator description:** Control group was assigned alongside two resistance-training periodization groups. The article describes the 16-week LP and UP resistance-training programs for the training arms; no structured training, education, placebo/sham, stretching, or other comparator activity was described for the control group during the treatment period.  **Original wording / direct quote:** “assigned into three groups … LP … UP … and a control group” / “After the familiarization period, participants initiated the LP and UP RT programs with two weekly sessions throughout the 16-week treatment period.”  **Source page/section:** Abstract; Methods; Participants; Resistance training | **Final comparator category:** Inactive control  **Active comparator?** No  **Included in active-comparator-exclusion sensitivity analysis?** Yes | **Rationale for classification:** Comparator was a non-training control group with no structured intervention reported during the 16-week treatment period.  **Notes / uncertainty:** Control protocol details are still not extensively described. All groups appear to have undergone baseline screening/testing, and participants completed familiarization before the training programs; however, the actual 16-week treatment protocols were LP and UP resistance training only. No active control content was reported for the control arm. |
| **No.** 31  **Study ID:** Tarazona2016  **Analysis-level ID(s):** Tarazona2016  **Linked intervention arms:** ME | **Comparator arm label:** Control / regular primary care  **Full-text comparator description:** Control group received no exercise training and attended the regular primary care program established by their center. Protein-calorie and vitamin D supplementation were controlled in both groups.  **Original wording / direct quote:** “The control group received no training and they attended the regular primary care program established by their center.”  **Source page/section:** Methods; Randomization | **Final comparator category:** Usual care / standard treatment  **Active comparator?** No  **Included in active-comparator-exclusion sensitivity analysis?** Yes | **Rationale for classification:** Comparator received regular primary care without the supervised multicomponent exercise program.  **Notes / uncertainty:** Not pure inactive control because participants attended regular primary care and nutritional/vitamin D supplementation was controlled. No structured exercise, health education, social activity, stretching comparator, or sham exercise was reported for the control arm. |
| **No.** 32  **Study ID:** Urzi2019  **Analysis-level ID(s):** Urzi2019  **Linked intervention arms:** RE | **Comparator arm label:** Control / no placebo or treatment  **Full-text comparator description:** Control group received no placebo or treatment during the 12-week elastic resistance training intervention period. Participants otherwise remained in the nursing-home setting with usual diet and health status monitoring.  **Original wording / direct quote:** “The control group did not receive any placebo or treatment.”  **Source page/section:** Methods; ERT; Diet control, health status, and adherence | **Final comparator category:** Inactive control  **Active comparator?** No  **Included in active-comparator-exclusion sensitivity analysis?** Yes | **Rationale for classification:** Comparator received no structured intervention, placebo, or active physical comparator.  **Notes / uncertainty:** Straightforward inactive/no-treatment control. Nursing-home diet and health monitoring were background conditions for all participants, not an added comparator intervention. |
| **No.** 33  **Study ID:** Vaughan2014  **Analysis-level ID(s):** Vaughan2014  **Linked intervention arms:** ME | **Comparator arm label:** Control / waiting-list usual activities  **Full-text comparator description:** Control group was waitlisted for the 16-week exercise program, asked to continue usual activities, and asked to refrain from more than 60 min of formal exercise each week during the waiting period.  **Original wording / direct quote:** “on a waiting list” / “continue with usual activities”  **Source page/section:** Methods; Intervention and comparator | **Final comparator category:** Inactive control  **Active comparator?** No  **Included in active-comparator-exclusion sensitivity analysis?** Yes | **Rationale for classification:** Comparator was a waiting-list/usual-activity control without structured exercise or active comparator content.  **Notes / uncertainty:** Control participants received 4-weekly phone contact for retention, but this was not a structured education, social, or exercise intervention. |
| **No.** 34  **Study ID:** Enette2020  **Analysis-level ID(s):** Enette2020  **Linked intervention arms:** AE | **Comparator arm label:** Controls / interactive information sessions  **Full-text comparator description:** Control group did not perform aerobic training; during the same 9-week period, controls participated in interactive information sessions once weekly.  **Original wording / direct quote:** “controls were engaged in interactive information sessions” / “once a week, controls attended sessions detailing the health benefits of physical activity in seniors”  **Source page/section:** Abstract; Materials and methods; Interactive information sessions | **Final comparator category:** Active non-exercise control  **Active comparator?** Yes  **Included in active-comparator-exclusion sensitivity analysis?** No | **Rationale for classification:** Comparator involved structured interactive information sessions without physical exercise.  **Notes / uncertainty:** The comparator had an active non-exercise contact component and was excluded from the active-comparator-exclusion sensitivity analysis. |
| **No.** 35  **Study ID:** Hvid2017  **Analysis-level ID(s):** Hvid2017  **Linked intervention arms:** RE | **Comparator arm label:** Control / no interventions  **Full-text comparator description:** Control group received no intervention during the 12-week period, while the training group performed progressive high-intensity power training.  **Original wording / direct quote:** “CG: no interventions”  **Source page/section:** Abstract; Methods; Participants | **Final comparator category:** Inactive control  **Active comparator?** No  **Included in active-comparator-exclusion sensitivity analysis?** Yes | **Rationale for classification:** Comparator received no structured intervention.  **Notes / uncertainty:** Straightforward no-intervention control. No usual-care protocol, health education, placebo, stretching, or sham physical comparator was reported. |
| **No.** 36  **Study ID:** Küster2017  **Analysis-level ID(s):** Küster2017  **Linked intervention arms:** ME | **Comparator arm label:** WLC / wait-list control  **Full-text comparator description:** Wait-list control condition. Participants were assigned to physical training, cognitive training, or wait-list control for 10 weeks.  **Original wording / direct quote:** “wait-list control condition”  **Source page/section:** Abstract; Materials and Methods; Study design | **Final comparator category:** Inactive control  **Active comparator?** No  **Included in active-comparator-exclusion sensitivity analysis?** Yes | **Rationale for classification:** Comparator was a wait-list control without structured intervention during the comparison period.  **Notes / uncertainty:** The linked analysis-level intervention was ME; comparator classification was based on the control condition and did not alter the exercise intervention coding. |
| **No.** 37  **Study ID:** Ledreux2019  **Analysis-level ID(s):** Ledreux2019  **Linked intervention arms:** ME | **Comparator arm label:** Active control / non-adaptive cognitive training  **Full-text comparator description:** Active control condition used non-adaptive Cogmed working-memory training. It was identical in instructions and time commitment to adaptive cognitive training, but task difficulty stayed low and constant.  **Original wording / direct quote:** “active control group” / “non-adaptive control training condition”  **Source page/section:** Abstract; Methods; Control condition | **Final comparator category:** Active non-exercise control  **Active comparator?** Yes  **Included in active-comparator-exclusion sensitivity analysis?** No | **Rationale for classification:** Comparator involved structured low-load computer-based cognitive activity without physical exercise.  **Notes / uncertainty:** This is not inactive control. It is an attention/time-matched active non-exercise comparator and should be excluded in Sensitivity 1. |
| **No.** 38  **Study ID:** Ruiz2015  **Analysis-level ID(s):** Ruiz2015  **Linked intervention arms:** RE | **Comparator arm label:** Standard-care control  **Full-text comparator description:** Standard-care control group was informed of the positive effects of regular physical activity and attended daily scheduled nursing-home mobility sessions consisting of small active and passive movements and gentle rhythmic stretches.  **Original wording / direct quote:** “standard-care group” / “daily scheduled sessions … of mobility exercises” / “small active and passive movements … gentle stretches”  **Source page/section:** Methods; Standard care group | **Final comparator category:** Active physical control  **Active comparator?** Yes  **Included in active-comparator-exclusion sensitivity analysis?** No | **Rationale for classification:** Comparator included routine mobility exercises and gentle stretching, which are physical activity components.  **Notes / uncertainty:** Although labelled standard care, usual care itself contained active/passive mobility and stretching sessions. Therefore classify as Active physical control rather than Usual care / standard treatment. |
| **No.** 39  **Study ID:** Vedovelli2017  **Analysis-level ID(s):** Vedovelli2017  **Linked intervention arms:** AE + RE | **Comparator arm label:** Control / no exercise  **Full-text comparator description:** Control condition received no exercise. Participants resided in the same retirement home and followed its usual routine, mainly determined by mealtimes.  **Original wording / direct quote:** “control condition (no exercise)”  **Source page/section:** Abstract; Methods; Sample | **Final comparator category:** Inactive control  **Active comparator?** No  **Included in active-comparator-exclusion sensitivity analysis?** Yes | **Rationale for classification:** Comparator received no structured exercise or active comparator intervention.  **Notes / uncertainty:** Usual institutional routine was background living condition, not an added comparator intervention. No education, social activity, placebo, stretching comparator, or usual-care treatment protocol was described. |
| **No.** 40  **Study ID:** Deus2021  **Analysis-level ID(s):** Deus2021  **Linked intervention arms:** RE | **Comparator arm label:** CTL / usual hemodialysis care  **Full-text comparator description:** Control group continued usual hemodialysis care and received the same general multidisciplinary recommendations as the RT group, but did not receive resistance training.  **Original wording / direct quote:** “allocated to the control group” / “received the same recommendations”  **Source page/section:** Abstract; Methods; Randomization; Resistance Training Protocol | **Final comparator category:** Usual care / standard treatment  **Active comparator?** No  **Included in active-comparator-exclusion sensitivity analysis?** Yes | **Rationale for classification:** Comparator received routine clinical hemodialysis care and general recommendations without the structured resistance training program.  **Notes / uncertainty:** Not pure inactive control because this is a clinical hemodialysis population under ongoing standard treatment and multidisciplinary recommendations. No additional exercise, sham, stretching, or active physical comparator was provided to CTL. |
| **No.** 41  **Study ID:** Byun2016  **Analysis-level ID(s):** Byun2016  **Linked intervention arms:** AE + RE | **Comparator arm label:** CG / Control group  **Full-text comparator description:** Control group was compared with the SBHE exercise group. Participants had no regular exercise experience in the previous 6 months; the study was conducted in an exercise group and a control group, and only the exercise group received the 12-week SBHE program. No structured comparator intervention was reported for CG.  **Original wording / direct quote:** “divided into the exercise group … and the control group” / “the elderlies who had no regular exercise experience in recent 6 months” / “The SBHE program was implemented 50 minutes daily, 4 times … weekly for 12 weeks”  **Source page/section:** Methods; Subjects; Senior brain health exercise program | **Final comparator category:** Inactive control  **Active comparator?** No  **Included in active-comparator-exclusion sensitivity analysis?** Yes | **Rationale for classification:** Comparator appears to be a non-exercise control arm, while the structured SBHE intervention was delivered only to the exercise group.  **Notes / uncertainty:** CG activities were not described in detail, so this remains a limited-description control. However, the paper does not report health education, social activity, placebo/sham, stretching, or any other active comparator content for CG. Retain Inactive control with this uncertainty note. |
| **No.** 42  **Study ID:** Chou2022  **Analysis-level ID(s):** Chou2022  **Linked intervention arms:** AE | **Comparator arm label:** Control / routine care  **Full-text comparator description:** Control group received routine care and a manual on healthy living. The manual covered topics such as nutrition, exercise, travel safety, age-appropriate preventive services, and legal/financial issues. Participants were encouraged to continue usual activity.  **Original wording / direct quote:** “control group with routine care” / “received routine care and a manual on healthy living” / “encouraged to continue engaging in their usual activity”  **Source page/section:** Abstract; Materials and Methods; Design and Participants | **Final comparator category:** Usual care / standard treatment  **Active comparator?** No  **Included in active-comparator-exclusion sensitivity analysis?** Yes | **Rationale for classification:** Comparator received routine care without the supervised aerobic walking intervention.  **Notes / uncertainty:** Not pure inactive control because routine care and a healthy-living manual were provided. However, this was not a structured health education class, attention-control session, social activity, or physical comparator; therefore classify as usual care rather than active non-exercise control. |
| **No.** 43  **Study ID:** Kim2018  **Analysis-level ID(s):** Kim2018  **Linked intervention arms:** AQE | **Comparator arm label:** Control group  **Full-text comparator description:** Control group did not receive the 16-week aquarobic exercise program. Participants were sedentary / not regularly physically active, and were advised to make no changes to their diet and exercise habits during the study.  **Original wording / direct quote:** “12 in the control group and 14 in the exercise group” / “Participants were advised to make no changes to their diet and exercise habits during the study.”  **Source page/section:** Materials and methods; Participants; Study design | **Final comparator category:** Inactive control  **Active comparator?** No  **Included in active-comparator-exclusion sensitivity analysis?** Yes | **Rationale for classification:** Comparator received no structured exercise intervention and maintained habitual diet and exercise habits.  **Notes / uncertainty:** No health education, usual-care protocol, placebo, stretching, sham exercise, or active comparator was reported for the control group. |
| **No.** 44  **Study ID:** Kang2020  **Analysis-level ID(s):** Kang2020  **Linked intervention arms:** AQE | **Comparator arm label:** Control group  **Full-text comparator description:** Control group did not receive the 16-week aquatic exercise program. Participants were sedentary and not regularly physically active; the control group was measured before and after the 16-week period and participants were advised not to change diet or exercise habits during the study.  **Original wording / direct quote:** “randomly … divided … into a control group … and an aquatic exercise group” / “All women were sedentary, did not regularly participate in physical activity” / “Participants were advised to make no changes to their diet and exercise habits during the study.”  **Source page/section:** Research method; Subjects; Study design | **Final comparator category:** Inactive control  **Active comparator?** No  **Included in active-comparator-exclusion sensitivity analysis?** Yes | **Rationale for classification:** Comparator received no structured aquatic exercise or other active comparator intervention and maintained habitual diet/exercise habits.  **Notes / uncertainty:** The paper explicitly states sedentary status and no change to diet/exercise habits during the study. No health education, usual-care protocol, placebo/sham, stretching comparator, or other active comparator content was reported for the control group. |
| **No.** 45  **Study ID:** Singsanan2024  **Analysis-level ID(s):** Singsanan2024  **Linked intervention arms:** MBE | **Comparator arm label:** CG / Control group  **Full-text comparator description:** Control group did not receive qigong training. The qigong group performed supervised qigong three times weekly for 8 weeks, whereas the control group was instructed to maintain regular behaviors and dietary habits during the same period.  **Original wording / direct quote:** “assigned to qigong exercise … or control … groups” / “The qigong group performed the qigong exercise three times per week, for 8 weeks.” / “Participants in the CG were instructed to sustain their regular behaviors and dietary habits throughout the 8 weeks.”  **Source page/section:** Abstract; Materials and Methods; Design; Qigong Training Intervention | **Final comparator category:** Inactive control  **Active comparator?** No  **Included in active-comparator-exclusion sensitivity analysis?** Yes | **Rationale for classification:** Comparator maintained usual behaviors and diet and did not receive structured qigong, exercise, placebo/sham activity, or other active comparator intervention.  **Notes / uncertainty:** The paper uses the phrase quasi-experimental/placebo-controlled design, but the actual comparator description available in the Methods indicates maintenance of regular behaviors and dietary habits; no concrete placebo/sham intervention is described for CG. Therefore, retain Inactive control rather than Active non-exercise control. |
| **No.** 46  **Study ID:** Solianik2021  **Analysis-level ID(s):** Solianik2021  **Linked intervention arms:** MBE | **Comparator arm label:** Control group / no intervention  **Full-text comparator description:** Control group received no tai chi intervention during the 10-week period and was asked to maintain daily routines and refrain from new exercise interventions.  **Original wording / direct quote:** “randomized to either a control group … or a tai chi group” / “The tai chi group received two, 8-form tai chi classes of 60 min duration per week.”  **Source page/section:** Abstract; Methods; Study design; Interventions | **Final comparator category:** Inactive control  **Active comparator?** No  **Included in active-comparator-exclusion sensitivity analysis?** Yes | **Rationale for classification:** Comparator received no structured intervention and maintained usual daily routines without new exercise.  **Notes / uncertainty:** Straightforward inactive/no-intervention control. No education, social activity, placebo, stretching, or sham physical comparator was reported. |
| **No.** 47  **Study ID:** Čekanauskaitė2020  **Analysis-level ID(s):** Čekanauskaitė2020  **Linked intervention arms:** MBE | **Comparator arm label:** Control group / daily living habits  **Full-text comparator description:** Control group was instructed to maintain daily living habits, and all participants were asked to refrain from sporting activities during the 10-week study period.  **Original wording / direct quote:** “participants … were instructed to maintain their daily living habits” / “All participants were asked to refrain from any sporting activities during the study.”  **Source page/section:** Material and methods; Study design | **Final comparator category:** Inactive control  **Active comparator?** No  **Included in active-comparator-exclusion sensitivity analysis?** Yes | **Rationale for classification:** Comparator maintained daily living habits and received no structured yoga, exercise, or active comparator intervention.  **Notes / uncertainty:** Straightforward inactive/usual-lifestyle control. No health education, social contact intervention, placebo/sham, stretching comparator, or active physical comparator was reported for the control group. |

**Note.** Comparator/control conditions were re-extracted from the full-text reports and classified according to the original comparator description. Active comparator conditions included active non-exercise controls and active physical controls. Studies without a non-exercise control arm were handled separately and retained their original exercise–exercise comparison structure; they were not treated as an additional comparator node. Counts are reported at the 47-study level. The Bo-Ram Kim source was represented by two analysis-level study IDs in the dataset but is listed as one study-level row here. Cell shading in the final-category column is used only as a visual aid: light blue indicates inactive control, light green indicates usual care/standard treatment, light yellow indicates active non-exercise control, light pink indicates active physical control, and light purple indicates no non-exercise control arm.

**Abbreviations.** AE, aerobic exercise; AE + RE, combined aerobic and resistance exercise; AQE, aquatic exercise; CE, coordination exercise; HIIT, high-intensity interval training; MBE, mind-body exercise; ME, mixed exercise; RE, resistance exercise; BDNF, brain-derived neurotrophic factor; CG, control group; mCIMT, modified constraint-induced movement therapy; MCI, mild cognitive impairment; NMA, network meta-analysis; PMR, progressive muscle relaxation.

### 4.4.4 Supplementary Table S4. Comparator-handling sensitivity analyses

| **Analysis** | **Parameter** | **Estimate** | **Lower 95% CrI** | **Upper 95% CrI** | **Interpretation** |
| --- | --- | --- | --- | --- | --- |
| **Primary analysis** | AE | 0.245 | 0.086 | 0.403 | Positive effect |
| **Primary analysis** | AE + RE | 0.579 | 0.276 | 0.877 | Positive effect |
| **Primary analysis** | AQE | 1.000 | 0.442 | 1.559 | Positive effect |
| **Primary analysis** | CE | 0.109 | -0.231 | 0.449 | Uncertain effect |
| **Primary analysis** | HIIT | 0.253 | -0.097 | 0.598 | Uncertain effect |
| **Primary analysis** | MBE | 0.612 | 0.342 | 0.883 | Positive effect |
| **Primary analysis** | ME | 0.305 | 0.135 | 0.475 | Positive effect |
| **Primary analysis** | RE | 0.374 | 0.213 | 0.534 | Positive effect |
| **Excluding active comparator conditions** | AE | 0.369 | 0.129 | 0.614 | Positive effect |
| **Excluding active comparator conditions** | AE + RE | 0.603 | 0.295 | 0.906 | Positive effect |
| **Excluding active comparator conditions** | AQE | 1.032 | 0.460 | 1.607 | Positive effect |
| **Excluding active comparator conditions** | CE | -0.012 | -0.421 | 0.400 | Uncertain effect |
| **Excluding active comparator conditions** | HIIT | 0.551 | 0.082 | 1.023 | Positive effect |
| **Excluding active comparator conditions** | MBE | 0.641 | 0.363 | 0.918 | Positive effect |
| **Excluding active comparator conditions** | ME | 0.526 | 0.306 | 0.746 | Positive effect |
| **Excluding active comparator conditions** | RE | 0.413 | 0.235 | 0.590 | Positive effect |
| **Separated comparator nodes** | Control: usual care / standard treatment | -0.088 | -0.326 | 0.151 | Uncertain effect |
| **Separated comparator nodes** | Control: active non-exercise | 0.243 | -0.009 | 0.499 | Uncertain effect |
| **Separated comparator nodes** | Control: active physical | 0.187 | -0.075 | 0.454 | Uncertain effect |
| **Separated comparator nodes** | AE | 0.335 | 0.135 | 0.537 | Positive effect |
| **Separated comparator nodes** | AE + RE | 0.613 | 0.312 | 0.916 | Positive effect |
| **Separated comparator nodes** | AQE | 1.029 | 0.461 | 1.603 | Positive effect |
| **Separated comparator nodes** | CE | 0.187 | -0.174 | 0.548 | Uncertain effect |
| **Separated comparator nodes** | HIIT | 0.325 | -0.039 | 0.687 | Uncertain effect |
| **Separated comparator nodes** | MBE | 0.641 | 0.364 | 0.918 | Positive effect |
| **Separated comparator nodes** | ME | 0.385 | 0.175 | 0.599 | Positive effect |
| **Separated comparator nodes** | RE | 0.370 | 0.157 | 0.584 | Positive effect |
| **Active comparator status as covariate** | AE | 0.255 | 0.083 | 0.427 | Positive effect |
| **Active comparator status as covariate** | AE + RE | 0.793 | 0.471 | 1.116 | Positive effect |
| **Active comparator status as covariate** | AQE | 1.001 | 0.433 | 1.566 | Positive effect |
| **Active comparator status as covariate** | CE | 0.045 | -0.471 | 0.566 | Uncertain effect |
| **Active comparator status as covariate** | HIIT | 0.248 | -0.108 | 0.599 | Uncertain effect |
| **Active comparator status as covariate** | MBE | 0.616 | 0.343 | 0.892 | Positive effect |
| **Active comparator status as covariate** | ME | 0.269 | 0.090 | 0.451 | Positive effect |
| **Active comparator status as covariate** | RE | 0.373 | 0.211 | 0.531 | Positive effect |
| **Active comparator status as covariate** | Active comparator status | 0.027 | -0.187 | 0.246 | Uncertain effect |

**Note.** Estimates are Hedges’ g with 95% credible intervals. In the separated-comparator-node analysis, the broad comparator node was separated into inactive control, usual care/standard treatment, active non-exercise control, and active physical control. Studies without a non-exercise control arm retained their original exercise–exercise comparison structure and were not treated as an additional comparator node. Comparator-node rows represent differences relative to inactive control; exercise rows represent intervention effects relative to inactive control. In the covariate model, “Active comparator status” represents the coefficient for active comparator conditions relative to non-active/usual-care comparator conditions. Positive effect was defined as a 95% CrI excluding zero in the positive direction.

### 4.4.5 Supplementary Table S5. Assessment of potential effect modifiers across comparator categories.

| **Comparator category** | **No. of R studyID** | **Mean age, median [IQR]** | **% female, median [IQR]** | **No. of exercise arms** | **Intervention duration, weeks, median [IQR]** | **Session duration, min, median [IQR]** | **Intensity, METs, median [IQR]** | **Weekly dose, MET-min/week, median [IQR]** | **Health status distribution** | **Supervision status distribution** |
| --- | --- | --- | --- | --- | --- | --- | --- | --- | --- | --- |
| Active non-exercise control | 8 | 68.1 [66.7, 74.4] | 67 [56.9, 76.1] | 9 | 12 [6, 12] | 50 [40, 60] | 5 [4, 5.2] | 720 [480, 750] | N: 4; Y: 4 | Y: 9 |
| Active physical control | 6 | 70.1 [67, 75.6] | 65.8 [43.6, 76.6] | 7 | 12 [10, 19] | 40 [35, 46.5] | 4.5 [3.8, 5] | 480 [360, 732.8] | N: 2; Y: 4 | Y: 7 |
| Inactive control | 26 | 68.9 [65.4, 71.6] | 100 [69.6, 100] | 29 | 12 [10, 12] | 60 [50, 60] | 4.8 [4, 5] | 600 [480, 855] | N: 9; Y: 17 | Y: 29 |
| No non-exercise control arm | 4 | 66.4 [61.9, 70.3] | 89 [67.6, 100] | 9 | 12 [8, 24] | 60 [40, 60] | 4.5 [3, 5] | 525 [360, 600] | N: 3; Y: 1 | Y: 9 |
| Usual care / standard treatment | 4 | 73.5 [68.9, 78.1] | 63.8 [51.8, 80.1] | 4 | 24 [24, 24] | 56 [46.5, 61.2] | 4 [3.9, 4.2] | 660 [541, 946.2] | N: 2; Y: 2 | Y: 4 |

**Note.** Values are presented as median [IQR] unless otherwise specified. Comparator categories were defined according to the full-text comparator descriptions. The number of R studyID reflects analysis-level study IDs; therefore, these counts may differ slightly from the 47-study-level counts reported in Supplementary Table S3. Studies without a non-exercise control arm were summarized separately because they did not contribute to the broad comparator/control node and retained their original exercise–exercise comparison structure. Y/N values in the health status and supervision columns follow the coding used in the original extraction dataset.

### 4.4.6 Supplementary Figure S6. Split-comparator network plot.


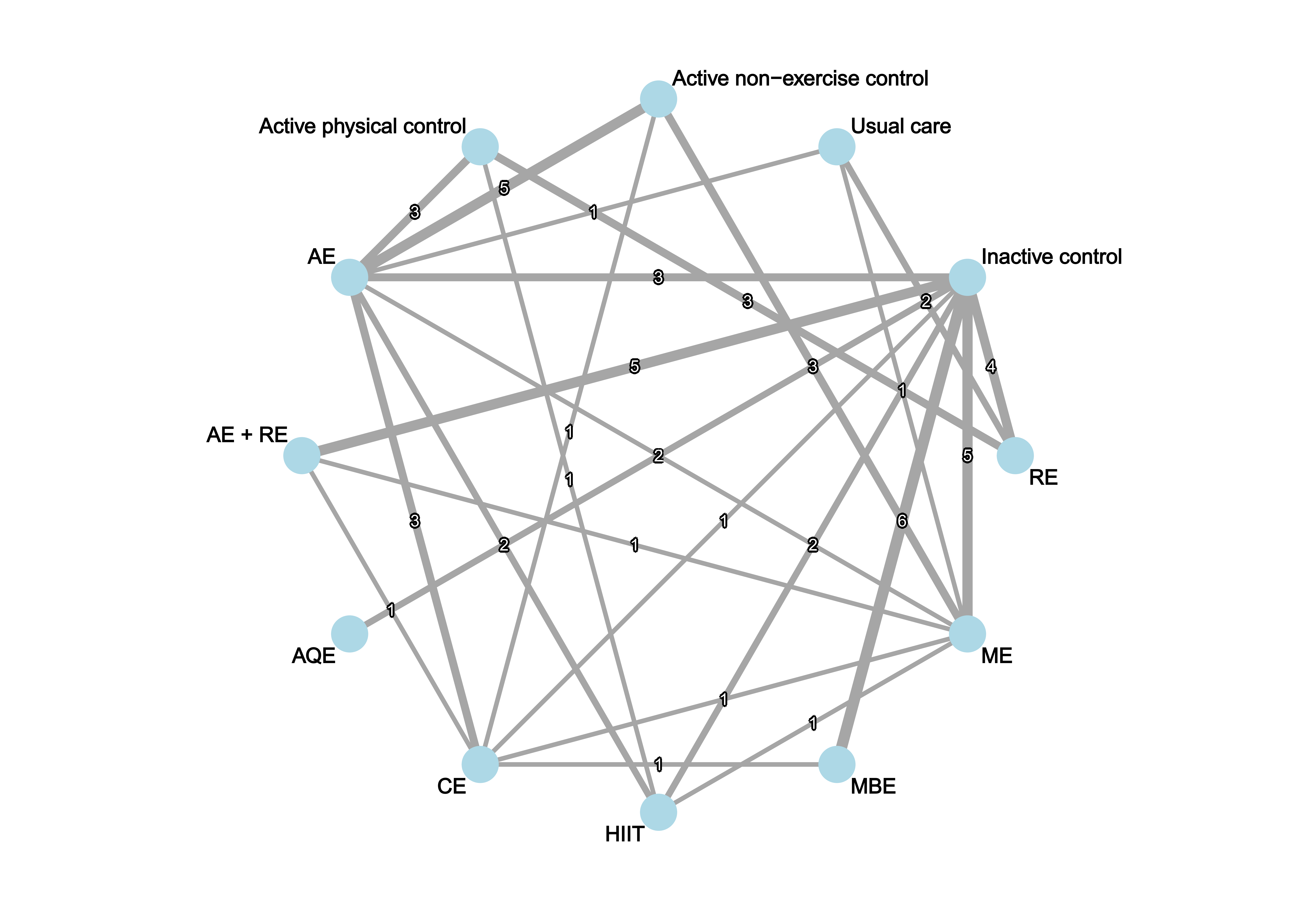


The broad comparator node was separated into four comparator-specific nodes: inactive control, usual care/standard treatment, active non-exercise control, and active physical control. Studies without a non-exercise control arm retained their original exercise–exercise comparison structure and were not treated as an additional comparator node. Line thickness indicates the number of direct comparisons.

# Appendix 5: Predicted responses

**Table 1. Predicted Effects of Exercise on BDNF**

| **weekly_dose** | **pred** | **se** | **lower** | **upper** |
| --- | --- | --- | --- | --- |
| 0 | 0.66251 | 0.33442 | 0.11243 | 1.21259 |
| 20 | 0.65363 | 0.32177 | 0.12437 | 1.18289 |
| 40 | 0.64474 | 0.3092 | 0.13616 | 1.15333 |
| 60 | 0.63586 | 0.29673 | 0.14779 | 1.12393 |
| 80 | 0.62698 | 0.28437 | 0.15923 | 1.09473 |
| 100 | 0.6181 | 0.27214 | 0.17046 | 1.06573 |
| 120 | 0.60921 | 0.26006 | 0.18145 | 1.03698 |
| 140 | 0.60033 | 0.24815 | 0.19216 | 1.0085 |
| 160 | 0.59145 | 0.23643 | 0.20255 | 0.98034 |
| 180 | 0.58256 | 0.22494 | 0.21257 | 0.95256 |
| 200 | 0.57368 | 0.21371 | 0.22217 | 0.92519 |
| 220 | 0.5648 | 0.20278 | 0.23126 | 0.89833 |
| 240 | 0.55592 | 0.19221 | 0.23977 | 0.87208 |
| 260 | 0.54709 | 0.18207 | 0.2476 | 0.84657 |
| 280 | 0.53833 | 0.17246 | 0.25465 | 0.822 |
| 300 | 0.5297 | 0.16347 | 0.26081 | 0.79858 |
| 320 | 0.52125 | 0.15518 | 0.266 | 0.7765 |
| 340 | 0.51306 | 0.14768 | 0.27016 | 0.75597 |
| 360 | 0.50518 | 0.14101 | 0.27324 | 0.73712 |
| 380 | 0.49767 | 0.13522 | 0.27525 | 0.72009 |
| 400 | 0.49059 | 0.13031 | 0.27625 | 0.70493 |
| 420 | 0.48399 | 0.12625 | 0.27633 | 0.69166 |
| 440 | 0.47793 | 0.12298 | 0.27564 | 0.68022 |
| 460 | 0.47244 | 0.12042 | 0.27436 | 0.67051 |
| 480 | 0.46756 | 0.11846 | 0.27271 | 0.66242 |
| 500 | 0.46333 | 0.117 | 0.27088 | 0.65577 |
| 520 | 0.45975 | 0.11591 | 0.2691 | 0.65041 |
| 540 | 0.45685 | 0.11511 | 0.26751 | 0.6462 |
| 560 | 0.45463 | 0.11452 | 0.26626 | 0.643 |
| 580 | 0.45307 | 0.11408 | 0.26543 | 0.64071 |
| 600 | 0.45216 | 0.11375 | 0.26505 | 0.63926 |
| 620 | 0.45187 | 0.11352 | 0.26515 | 0.6386 |
| 640 | 0.4522 | 0.11339 | 0.26569 | 0.63872 |
| 660 | 0.45311 | 0.11338 | 0.26662 | 0.6396 |
| 680 | 0.45456 | 0.11352 | 0.26784 | 0.64128 |
| 700 | 0.45652 | 0.11384 | 0.26926 | 0.64378 |
| 720 | 0.45894 | 0.1144 | 0.27076 | 0.64712 |
| 740 | 0.46176 | 0.11524 | 0.27221 | 0.65132 |
| 760 | 0.46494 | 0.1164 | 0.27349 | 0.65639 |
| 780 | 0.4684 | 0.11789 | 0.27449 | 0.66232 |
| 800 | 0.47209 | 0.11974 | 0.27513 | 0.66904 |
| 820 | 0.47593 | 0.12194 | 0.27535 | 0.67651 |
| 840 | 0.47986 | 0.12448 | 0.27512 | 0.6846 |
| 860 | 0.48382 | 0.1273 | 0.27443 | 0.69322 |
| 880 | 0.48776 | 0.13039 | 0.27328 | 0.70223 |
| 900 | 0.49159 | 0.13368 | 0.27171 | 0.71148 |
| 920 | 0.49528 | 0.13713 | 0.26973 | 0.72083 |
| 940 | 0.49875 | 0.14068 | 0.26736 | 0.73014 |
| 960 | 0.50196 | 0.14429 | 0.26462 | 0.7393 |
| 980 | 0.50485 | 0.14794 | 0.26152 | 0.74819 |
| 1000 | 0.50738 | 0.15159 | 0.25804 | 0.75672 |
| 1020 | 0.50951 | 0.15524 | 0.25416 | 0.76486 |
| 1040 | 0.51121 | 0.15891 | 0.24983 | 0.7726 |
| 1060 | 0.51248 | 0.16264 | 0.24497 | 0.77999 |
| 1080 | 0.5133 | 0.16646 | 0.2395 | 0.7871 |
| 1100 | 0.51368 | 0.17045 | 0.23331 | 0.79405 |
| 1120 | 0.51363 | 0.17468 | 0.2263 | 0.80096 |
| 1140 | 0.51317 | 0.17923 | 0.21837 | 0.80797 |
| 1160 | 0.51231 | 0.18416 | 0.20939 | 0.81523 |
| 1180 | 0.51107 | 0.18956 | 0.19926 | 0.82287 |
| 1200 | 0.50947 | 0.19549 | 0.18791 | 0.83102 |
| 1220 | 0.50751 | 0.20201 | 0.17524 | 0.83979 |
| 1240 | 0.50523 | 0.20916 | 0.1612 | 0.84926 |
| 1260 | 0.50263 | 0.21697 | 0.14575 | 0.85952 |
| 1280 | 0.49974 | 0.22548 | 0.12887 | 0.87061 |
| 1300 | 0.49656 | 0.23468 | 0.11054 | 0.88258 |
| 1320 | 0.49312 | 0.2446 | 0.09079 | 0.89545 |
| 1340 | 0.48942 | 0.25521 | 0.06963 | 0.90922 |
| 1360 | 0.4855 | 0.26651 | 0.04712 | 0.92387 |
| 1380 | 0.48135 | 0.27847 | 0.02331 | 0.9394 |
| 1400 | 0.47701 | 0.29107 | -0.00175 | 0.95578 |
| 1420 | 0.47248 | 0.30426 | -0.02799 | 0.97295 |
| 1440 | 0.46779 | 0.31803 | -0.05532 | 0.9909 |
| 1460 | 0.46294 | 0.33232 | -0.08367 | 1.00956 |
| 1480 | 0.45796 | 0.3471 | -0.11296 | 1.02888 |
| 1500 | 0.45286 | 0.36233 | -0.14311 | 1.04883 |

# Appendix 6: Included randomized controlled trials contributing data to the meta-analysis

1. Tait JL, Duckham RL, Rantalainen T, Milte CM, Main LC, Nowson CA, et al. Effects of a 6-month dual-task, power-based exercise program on cognitive function, neurological and inflammatory markers in older adults: secondary analysis of a cluster randomised controlled trial. GeroScience. 2025;47(1):1251-68.
2. Baharavar F, Vakili J, Sari-Sarraf V, Pourmanaf H. The effect of circuit training on muscle synthesis’s indices, neurotrophic factors, and physical fitness in elderly women: a randomized controlled trial. Sport Sciences for Health. 2025:1-8.
3. De Sá CA, Saretto CB, Cardoso AM, Remor A, Breda CO, da Silva Corralo V. Effects of a physical exercise or motor activity protocol on cognitive function, lipid profile, and BDNF levels in older adults with mild cognitive impairment. Molecular and Cellular Biochemistry. 2024;479(3):499-509.
4. Salisbury DL, Li D, Todd M, Ng TK, Yu F. Aerobic Exercise, Training Dose, and Cardiorespiratory Fitness: Effects and Relationships with Resting Plasma Neurotrophic Factors in Alzheimer’s Dementia. Journal of vascular diseases. 2023;2(3):351-66.
5. Zhang Q, Zhu M, Huang L, Zhu M, Liu X, Zhou P, et al. A study on the effect of traditional Chinese exercise combined with rhythm training on the intervention of older adults with mild cognitive impairment. American Journal of Alzheimer's Disease & Other Dementias®. 2023;38:15333175231190626.
6. Hola V, Polanska H, Jandova T, Jaklová Dytrtová J, Weinerova J, Steffl M, et al. The Effect of Two Somatic-Based Practices Dance and Martial Arts on Irisin, BDNF Levels and Cognitive and Physical Fitness in Older Adults: A Randomized Control Trial. Clinical Interventions in Aging. 2024:1829-42.
7. Rahmawati NR, Tinduh D, Utami DA, Utomo B. Effect of boxing exergame addition on brain-derived neurotrophic factor of elderly women. Romanian JouRnal of neuRology. 2024;23(2):154.
8. García-Salazar LF, Pereira ND, Silva ESM, Ribeiro JAM, Nagai Ocamoto G, Mendes Zambetta R, et al. Could aerobic exercise applied before constraint-induced movement therapy change circulating molecular biomarkers in chronic post-stroke? Physiotherapy Theory and Practice. 2024:1-12.
9. Freeberg KA, Craighead DH, Heinbockel TC, Rossman MJ, Jackman RA, Jankowski LR, et al. Time-efficient, high-resistance inspiratory muscle strength training increases cerebrovascular reactivity in midlife and older adults. American Journal of Physiology-Heart and Circulatory Physiology. 2023;325(5):H1059-H68.
10. Khanthong P, Sriyakul K, Dechakhamphu A, Krajarng A, Kamalashiran C, Jayathavaj V, et al. A randomized controlled trial on the effects of traditional Thai mind-body exercise (Ruesi Dadton) on biomarkers in mild cognitive impairment. European Journal of Physical and Rehabilitation Medicine. 2024;60(4):604.
11. Ghodrati N, Haghighi AH, Kakhak SAH, Abbasian S, Goldfield GS. Effect of combined exercise training on physical and cognitive function in women with type 2 diabetes. Canadian Journal of Diabetes. 2023;47(2):162-70.
12. Donyaei A, Kiani E, Bahrololoum H, Moser O. Effect of combined aerobic–resistance training and subsequent detraining on brain‐derived neurotrophic factor (BDNF) and depression in women with type 2 diabetes mellitus: A randomized controlled trial. Diabetic Medicine. 2024;41(3):e15188.
13. Rodziewicz-Flis EA, Kawa M, Kaczor JJ, Szaro-Truchan M, Flis DJ, Lombardi G, et al. Changes in selected exerkines concentration post folk-dance training are accompanied by glucose homeostasis and physical performance improvement in older adults. Scientific Reports. 2023;13(1):8596.
14. Tsai C-L. Acute effects of high-intensity interval exercise plus whole-body vibration on bone turnover markers, BDNF, irisin, and neurocognitive performance in postmenopausal women. Biological Psychology. 2025;196:109029.
15. de Souza HCM, Pessoa MF, dos Santos Clemente R, da Silva AV, Cardoso PRG, Fernandes J, et al. Effects of 12 weeks of inspiratory muscle training and whole body vibration on the inflammatory profile, BDNF and muscular system in pre-frail elderly women: A randomized controlled trial. Archives of Gerontology and Geriatrics. 2024;123:105421.
16. Maguire C, Betschart M, Pohl J, Primani F, Taeymans J, Hund-Georgiadis M. Effects of moderate-intensity aerobic exercise on serum BDNF and motor learning in the upper-limb in patients after chronic-stroke: A randomized, controlled feasibility study with embedded health economic evaluation. NeuroRehabilitation. 2023;52(3):485-506.
17. Arrieta H, Rezola-Pardo C, Kortajarena M, Hervás G, Gil J, Yanguas JJ, et al. The impact of physical exercise on cognitive and affective functions and serum levels of brain-derived neurotrophic factor in nursing home residents: a randomized controlled trial. Maturitas. 2020;131:72-7.
18. Damirchi A, Hosseini F, Babaei P. Mental training enhances cognitive function and BDNF more than either physical or combined training in elderly women with MCI: a small-scale study. American Journal of Alzheimer's Disease & Other Dementias®. 2018;33(1):20-9.
19. rickson KI, Voss MW, Prakash RS, Basak C, Szabo A, Chaddock L, et al. Exercise training increases size of hippocampus and improves memory. Proceedings of the national academy of sciences. 2011;108(7):3017-22.
20. Fragala MS, Beyer KS, Jajtner AR, Townsend JR, Pruna GJ, Boone CH, et al. Resistance exercise may improve spatial awareness and visual reaction in older adults. The Journal of Strength & Conditioning Research. 2014;28(8):2079-87.
21. Heissel A, Vesterling A, White SA, Kallies G, Behr D, Arafat AM, et al. Feasibility of an exercise program for older depressive inpatients. GeroPsych. 2015.
22. Kim B-R, Lim S-T, editors. Effects of leisure-time physical activity on cognitive reserve biomarkers and leisure motivation in the pre-diabetes elderly. Healthcare; 2022: MDPI.
23. Kohanpour M-A, Peeri M, Azarbayjani M-A. The effects of aerobic exercise with lavender essence use on cognitive state and serum brain-derived neurotrophic factor levels in elderly with mild cognitive impairment. Journal of herbmed pharmacology. 2017;6(2):80-4.
24. Kovacevic A, Fenesi B, Paolucci E, Heisz JJ. The effects of aerobic exercise intensity on memory in older adults. Applied physiology, nutrition, and metabolism. 2020;45(6):591-600.
25. Li X, Han T, Zou X, Zhang H, Feng W, Wang H, et al. Long-term high-intensity interval training increases serum neurotrophic factors in elderly overweight and obese Chinese adults. European Journal of Applied Physiology. 2021;121:2773-85.
26. Maass A, Düzel S, Brigadski T, Goerke M, Becke A, Sobieray U, et al. Relationships of peripheral IGF-1, VEGF and BDNF levels to exercise-related changes in memory, hippocampal perfusion and volumes in older adults. Neuroimage. 2016;131:142-54.
27. Matura S, Fleckenstein J, Deichmann R, Engeroff T, Füzéki E, Hattingen E, et al. Effects of aerobic exercise on brain metabolism and grey matter volume in older adults: results of the randomised controlled SMART trial. Translational psychiatry. 2017;7(7):e1172-e.
28. Manuela Crispim Nascimento C, Rodrigues Pereira J, Pires de Andrade L, Garuffi M, Leme Talib L, Vicente Forlenza O, et al. Physical exercise in MCI elderly promotes reduction of pro-inflammatory cytokines and improvements on cognition and BDNF peripheral levels. Current Alzheimer Research. 2014;11(8):799-805.
29. Osali A. Aerobic exercise and nano-curcumin supplementation improve inflammation in elderly females with metabolic syndrome. Diabetology & Metabolic Syndrome. 2020;12:1-7.
30. Prestes J, da Cunha Nascimento D, Tibana RA, Teixeira TG, Vieira DCL, Tajra V, et al. Understanding the individual responsiveness to resistance training periodization. Age. 2015;37:1-13.
31. Tarazona-Santabalbina FJ, Gómez-Cabrera MC, Pérez-Ros P, Martínez-Arnau FM, Cabo H, Tsaparas K, et al. A multicomponent exercise intervention that reverses frailty and improves cognition, emotion, and social networking in the community-dwelling frail elderly: a randomized clinical trial. Journal of the American Medical Directors Association. 2016;17(5):426-33.
32. Urzi F, Marusic U, Ličen S, Buzan E. Effects of elastic resistance training on functional performance and myokines in older women—a randomized controlled trial. Journal of the American Medical Directors Association. 2019;20(7):830-4. e2.
33. Vaughan S, Wallis M, Polit D, Steele M, Shum D, Morris N. The effects of multimodal exercise on cognitive and physical functioning and brain-derived neurotrophic factor in older women: a randomised controlled trial. Age and ageing. 2014;43(5):623-9.
34. Enette L, Vogel T, Merle S, Valard-Guiguet A-G, Ozier-Lafontaine N, Neviere R, et al. Effect of 9 weeks continuous vs. interval aerobic training on plasma BDNF levels, aerobic fitness, cognitive capacity and quality of life among seniors with mild to moderate Alzheimer’s disease: a randomized controlled trial. European Review of Aging and Physical Activity. 2020;17:1-16.
35. Hvid LG, Nielsen MK, Simonsen C, Andersen M, Caserotti P. Brain-derived neurotrophic factor (BDNF) serum basal levels is not affected by power training in mobility-limited older adults—a randomized controlled trial. Experimental gerontology. 2017;93:29-35.
36. Küster OC, Laptinskaya D, Fissler P, Schnack C, Zügel M, Nold V, et al. Novel blood-based biomarkers of cognition, stress, and physical or cognitive training in older adults at risk of dementia: preliminary evidence for a role of BDNF, irisin, and the kynurenine pathway. Journal of Alzheimer’s disease. 2017;59(3):1097-111.
37. Ledreux A, Håkansson K, Carlsson R, Kidane M, Columbo L, Terjestam Y, et al. Differential effects of physical exercise, cognitive training, and mindfulness practice on serum BDNF levels in healthy older adults: a randomized controlled intervention study. Journal of Alzheimer’s disease. 2019;71(4):1245-61.
38. Ruiz JR, Gil-Bea F, Bustamante-Ara N, Rodríguez-Romo G, Fiuza-Luces C, Serra-Rexach JA, et al. Resistance training does not have an effect on cognition or related serum biomarkers in nonagenarians: a randomized controlled trial. International journal of sports medicine. 2015;36(01):54-60.
39. Vedovelli K, Giacobbo BL, Corrêa MS, Wieck A, Argimon IIdL, Bromberg E. Multimodal physical activity increases brain-derived neurotrophic factor levels and improves cognition in institutionalized older women. Geroscience. 2017;39:407-17.
40. Deus LA, Corrêa HdL, Neves RVP, Reis AL, Honorato FS, Silva VL, et al. Are resistance training-induced BDNF in hemodialysis patients associated with depressive symptoms, quality of life, antioxidant capacity, and muscle strength? An insight for the muscle–brain–renal axis. International journal of environmental research and public health. 2021;18(21):11299.
41. Byun J-E, Kang E-B. The effects of senior brain health exercise program on basic physical fitness, cognitive function and BDNF of elderly women-a feasibility study. Journal of Exercise Nutrition & Biochemistry. 2016;20(2):8.
42. Chou C-C, Chien L-Y, Lin M-F, Wang C-J, Liu P-Y. Effects of Aerobic Walking on Memory, Subjective Cognitive Complaints, and Brain-Derived Neurotrophic Factor Among Older Hypertensive Women. Biological Research For Nursing. 2022;24(4):484-92.
43. Kim J-H, Kim D-Y. Aquarobic exercises improve the serum blood irisin and brain-derived neurotrophic factor levels in elderly women. Experimental gerontology. 2018;104:60-5.
44. Kang D-w, Bressel E, Kim D-y. Effects of aquatic exercise on insulin-like growth factor-1, brain-derived neurotrophic factor, vascular endothelial growth factor, and cognitive function in elderly women. Experimental gerontology. 2020;132:110842.
45. Singsanan S, Luangpon N, Kiatkulanusorn S, Boonsiri P, Burtscher M, Klarod K. Qigong Training Effects on Brain-Derived Neurotrophic Factor and Cognitive Functions in Sedentary Middle-Aged and Elderly Females With Type 2 Diabetes. Women in Sport and Physical Activity Journal. 2024;32(1).
46. Solianik R, Mickevičienė D, Žlibinaitė L, Čekanauskaitė A. Tai chi improves psychoemotional state, cognition, and motor learning in older adults during the COVID-19 pandemic. Experimental Gerontology. 2021;150:111363.
47. Čekanauskaitė A, Skurvydas A, Žlibinaitė L, Mickevičienė D, Kilikevičienė S, Solianik R. A 10-week yoga practice has no effect on cognition, but improves balance and motor learning by attenuating brain-derived neurotrophic factor levels in older adults. Experimental gerontology. 2020;138:110998.
